# Molecular basis and biological relevance of bacterial and plant pinoresinol/lariciresinol reductase specificities

**DOI:** 10.1002/pro.70436

**Published:** 2026-01-20

**Authors:** Clyde A. Smith, Diana L. Bedgar, Michael A. Costa, Syed G. A. Moinuddin, Marco N. Allemann, Joshua K. Michener, Laurence B. Davin, Norman G. Lewis

**Affiliations:** ^1^ Stanford Synchrotron Radiation Lightsource Menlo Park California USA; ^2^ Department of Chemistry Stanford University Stanford California USA; ^3^ Institute of Biological Chemistry Washington State University Pullman Washington USA; ^4^ Biosciences Division Oak Ridge National Laboratory Oak Ridge Tennessee USA

**Keywords:** Boltz‐2 molecular modeling, catabolism, lignans, lignin, lignin biodegradation, *Novosphingobium aromaticivorans* F199, *Novosphingobium rhizosphaerae*, pinoresinol lariciresinol reductase, pinoresinol reductase, *Sphingobium lignivorans* SYK‐6, western red cedar (*Thuja plicata*)

## Abstract

A bacterial pinoresinol/lariciresinol reductase (PLR) homolog named NrPinZ was obtained from a *Novosphingobium rhizosphaerae* sp. LY bacterial strain, with NrPinZ being part of its 5‐step biochemical system catabolizing pinoresinol into coniferyl aldehyde and vanillin. Recombinant NrPinZ reduces racemic 8–8′ furanofuran lignans [(±)‐pinoresinols, medioresinols, and syringaresinols] with similar overall catalytic efficiencies. In those reductions, only one of the two furan ring systems is reduced. Two other bacterial PLR homologs, NaPinZ and SlPinZ, from *N. aromaticivorans* F199 and *Sphingobium lignivorans* SYK‐6, respectively, had comparable substrate versatilities and catalytic efficacies. Plant PLR homologs, by comparison, are either enantiospecific, enantioselective, or variants thereof, being able to reduce either one or both furan rings. For example, a recombinant enantioselective PLR (PLR_Tp2) from western red cedar (*Thuja plicata*) preferentially reduces both (+)‐pinoresinol furan rings to afford (−)‐secoisolariciresinol. BoltZ‐2 modeling of NrPinZ and PLR_Tp2, together with substrate docking of (+)‐ and (−)‐pinoresinols, medioresinols, and syringaresinols, was very instructive. The NrPinZ active site P1/P2 sub‐pockets allow for both racemic forms to be catabolized. Conversely, the smaller P1 pocket in PLR_Tp2 preferentially positions (+)‐pinoresinol for downstream metabolism into (−)‐secoisolariciresinol, thereby providing a biochemical explanation for the different stereochemical outcomes. NrPinZ, NaPinZ, and SlPinZ, catalyzing substrate versatile catabolism of both racemic forms, may have important ramifications for gymnosperm and angiosperm lignin and lignan biodegradation, including its evolutionary significance and potential in enzyme engineering.

## 1 INTRODUCTION

Vascular plant lignans and polymeric lignins have important plant defense, medicinal, health protection, and indispensable vascular cell wall reinforcement properties *in planta* (Cesarino, 2019; Laskar et al., 2010; Lewis et al., 1999; Lewis & Davin, 1999; Vassão et al., 2010). Polymeric lignins also contribute greatly to lignocellulosic biomass recalcitrance (Bomble et al., 2017), and their presence hugely hinders facile production of lignocellulosic‐derived specialty/commodity chemicals and renewable biofuels, at a scale and cost‐competitiveness with petroleum and petrochemical products. This recalcitrance is largely due to (bio)chemically stable carbon–carbon (C–C) linkages in various lignin substructures, rather than the more readily cleavable carbon–oxygen (C–O) bonds.

**FIGURE 1:**
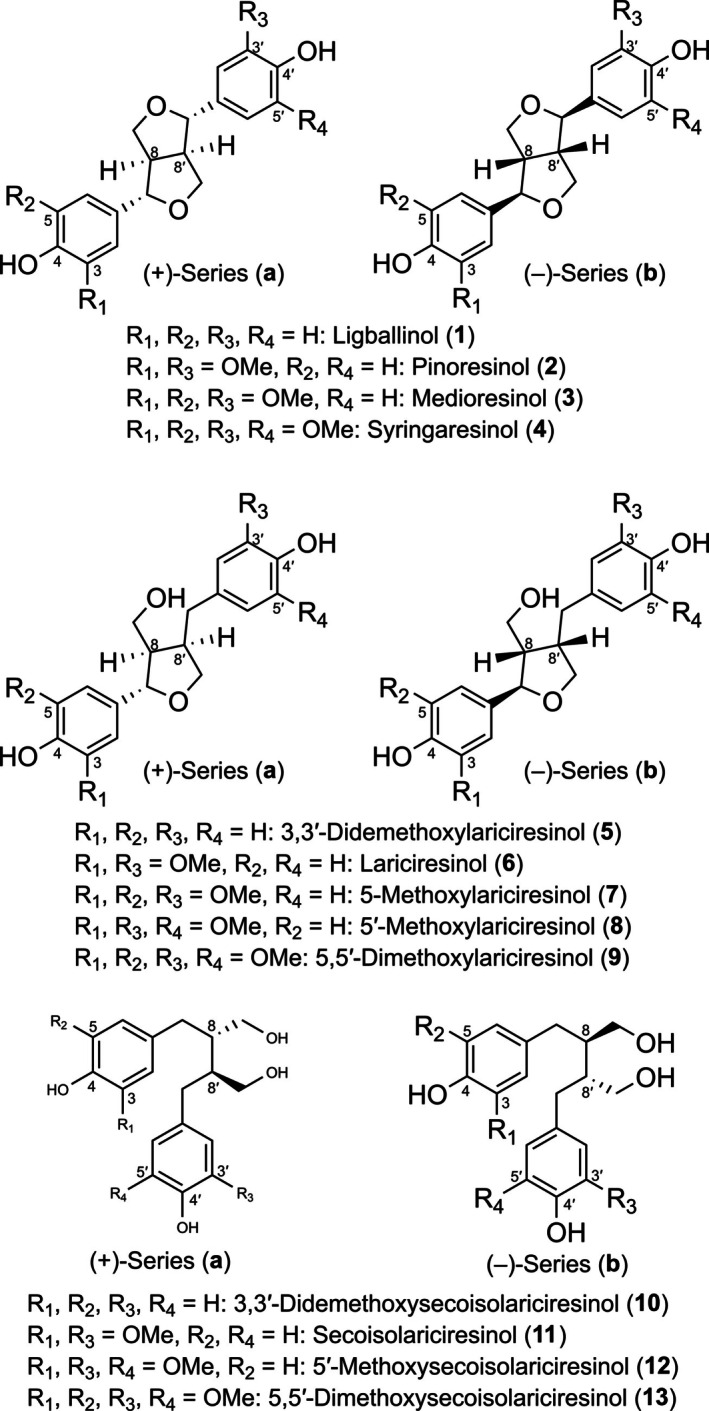
Furanofuran lignans (**1–4**), furanolignans (**5–9**), and dibenzylbutane lignans (**10–13**). “**a**” and “**b**” refer to the (+)‐ and (−)‐enantiomeric forms.

One such recalcitrant C–C linkage type is that found in racemic 8–8′ linked lignin sub‐structures and in 8–8′ linked lignans. By contrast, the latter can be found in either enantiomerically pure form or in varying amounts of isomers, including racemates. Examples include the four 8–8′ furanofuran lignan homologs, (+)‐ and/or (−)‐isomers of pinoresinol (**2a/b**), medioresinol (**3a/b**), syringaresinol (**4a/b**), and ligballinol (**1a/b**) in various vascular plant species (Figure 1) (Casabuono & Pomilio, 1994; Deyama, 1983; Kobayashi & Ohta, 1983; Lewis et al., 2022; Lin et al., 1994). They result from the coupling of different types of monomers, *p*‐coumaryl, coniferyl, 5‐hydroxyconiferyl, and sinapyl alcohols. These monomers are also differentially found in lignins throughout the vascular plant kingdom (including ferns, gymnosperms, and angiosperms). Of these, the more highly oxygenated sinapyl alcohol‐derived moiety is essentially found in angiosperms.

Recently, a bacterial strain described as *Novosphingobium rhizosphaerae* sp. LY (thereafter *N. rhizosphaerae*) isolated from a hot spring in Yellowstone National Park was found to be able to grow with a (+)‐pinoresinol (**2a**) enriched (*circa* 88% enantiomeric excess, e.e.) preparation as sole carbon source (Allemann et al., 2025); hereafter, this preparation is referred to as (+)‐pinoresinol (**2a**). In addition to a previously described pathway in *Sphingobium lignivorans* SYK‐6 on (±)‐pinoresinol (**2a/b**) and (±)‐syringaresinol (**4a/b**) catabolism (Fukuhara et al., 2013), *N. rhizosphaerae* was shown to catabolize (+)‐pinoresinol (**2a**) into vanillin and coniferyl aldehyde via a 5‐step enzymatic catabolism pathway. The first step in that biochemical sequence is catalyzed by a NADPH‐dependent pinoresinol reductase (PR) homolog, named NrPinZ, converting (±)‐pinoresinols (**2a/b**) into (±)‐lariciresinols (**6a/b**) (Allemann et al., 2025).

**FIGURE 2:**
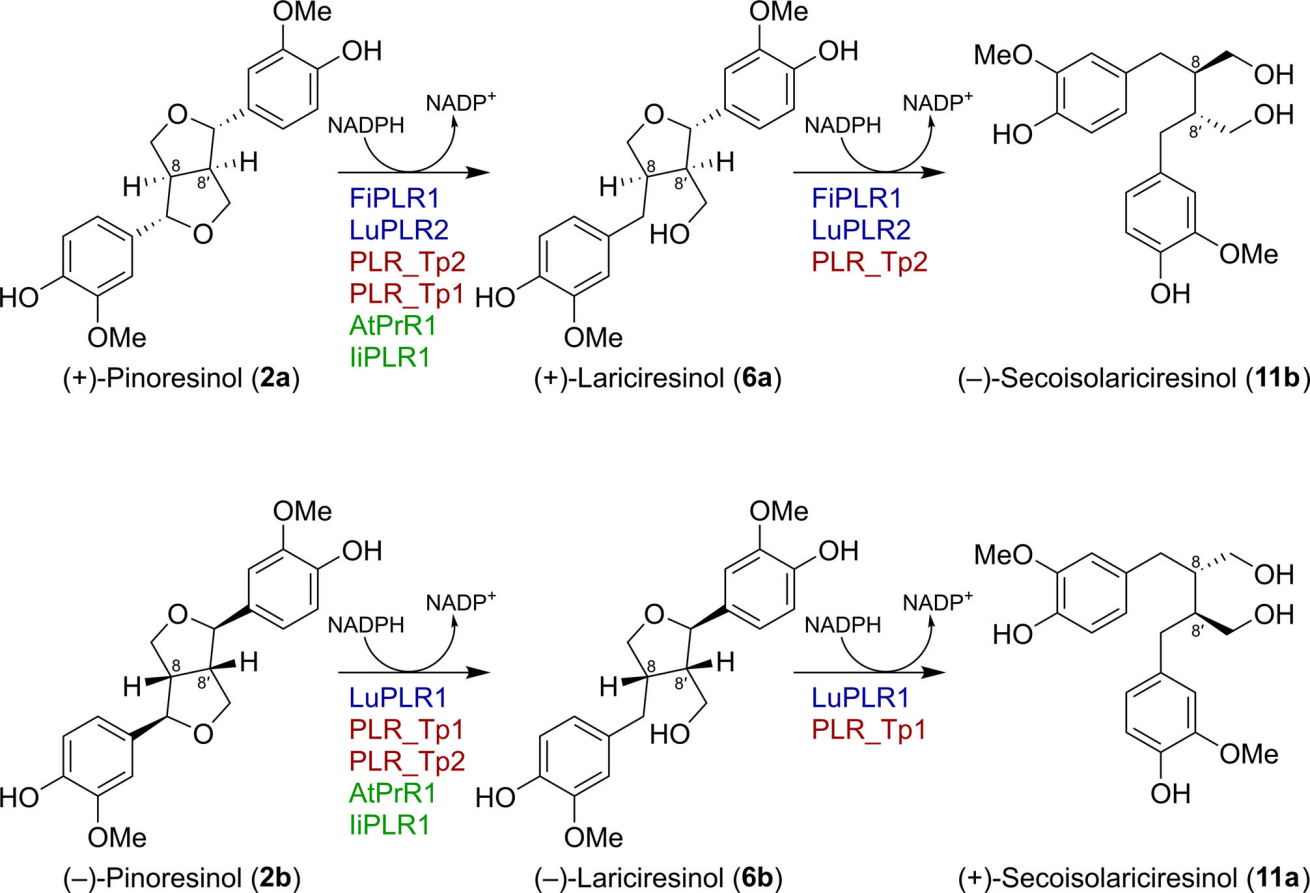
Reactions catalyzed *in vitro* by recombinant vascular plant pinoresinol/lariciresinol reductases (PLRs), pinoresinol reductases (PRs), and homologs thereof. At, *Arabidopsis thaliana*; Fi, *Forsythia intermedia*; Ii, *Isatis indigotica*; Lu, *Linum usitatissimum*; Tp, *Thuja plicata*. “**a**” and “**b**” refer to formation of the (+)‐ and (−)‐enantiomeric forms, respectively.

In much earlier work with Forsythia (*Forsythia intermedia*), we had discovered (and named) a vascular plant pinoresinol/lariciresinol reductase (PLR) PLR_Fi1 (Dinkova‐Kostova et al., 1996) that was able to enantiospecifically convert only (+)‐pinoresinol (**2a**) into (−)‐secoisolariciresinol (**11b**) via (+)‐lariciresinol (**6a**) (Figure 2). Flax (*Linum usitatissimum*) LuPLR2 (Hemmati et al., 2010) was subsequently shown to catalyze the same enantiospecific reaction. Both PLRs utilize (+)‐pinoresinol (**2a**) and (+)‐lariciresinol (**6a**) as substrates, being unable to reduce the corresponding antipodes. In an analogous manner, flax LuPLR1 enantiospecifically utilizes (−)‐pinoresinol (**2b**) to afford (+)‐secoisolariciresinol (**11a**) (Hemmati et al., 2010; von Heimendahl et al., 2005), but cannot process either (+)‐pinoresinol (**2a**) or (+)‐lariciresinol (**6a**).

Four other – albeit biochemically differing – plant PR/PLR homolog conversions are exemplified with AtPrR1 from *Arabidopsis thaliana* (Nakatsubo et al., 2008), PLR_Tp1 and PLR_Tp2 from western red cedar (*Thuja plicata*) (Fujita et al., 1999; Hwang et al., 2020), and IiPLR1 from *Isatis indigotica* (Chen et al., 2024; Xiao et al., 2015; Xiao et al., 2021). Of these, AtPrR1 converts racemic pinoresinols (**2a/b**) into (+)‐ and (−)‐lariciresinols (**6a/b**) (Figure 2), with the (+)‐enantiomer in 6% enantiomeric excess (e.e.); neither product is processed further (Nakatsubo et al., 2008). *k*_cat_/*K*_m_ values for both isolated enantiomers are very similar, 1.3 × 10^4^ and 1.9 × 10^4^ M^−1^ s^−1^ for (+)‐ and (−)‐pinoresinols (**2a** and **2b**), respectively (Table 1). By contrast, the PLR_Tp1 homolog more slowly converts isolated pinoresinol enantiomers (**2a**) and (**2b**) into lariciresinols (**6a** and **6b**), but only the (−)‐lariciresinol (**6b**) enantiomer is slowly further reduced to (+)‐secoisolariciresinol (**11a**) (*k*_cat_/*K*_m_ 8.1 × 10^3^ M^−1^ s^−1^) (Fujita et al., 1999; Hwang et al., 2020). PLR_Tp2 does the opposite from an enantiospecific perspective and acts much more efficiently on the isolated (+)‐pinoresinol (**2a**) enantiomer to afford (−)‐secoisolariciresinol (**11b**) (*k*_cat_/*K*_m_ 1.19 × 10^6^ M^−1^ s^−1^), but can also slowly reduce the isolated (−)‐pinoresinol (**2b**) enantiomer to give (−)‐lariciresinol (**6b**) (*k*_cat_/*K*_m_ 1.7 × 10^5^ M^−1^ s^−1^), as a final additional product under the conditions employed (Fujita et al., 1999; Hwang et al., 2020).

**TABLE 1:** Kinetic data for bacterial and vascular plant PLR/PR homologs.

Enzyme	Substrate	*K*_m_ (μM)	*V*_max_ (pkat/μg protein)	*k*_cat_ (s^−1^)	*k*_cat_/*K*_m_ (M^−1^ s^−1^)	Reference
*Recombinant proteins*						
NrPinZ	(±)‐Pinoresinols (**2a/b**)	27.0 ± 6.6	361.8 ± 22.4	12.7	4.69 × 10^5^	Allemann et al. (2025)
NrPinZ	(+)‐Pinoresinol (**2a**)	22.1 ± 3.2	307.5 ± 14.6	10.8	4.86 × 10^5^	Allemann et al. (2025)
NrPinZ	(±)‐Medioresinols (**3a/b**)	30.9 ± 8.7	383.8 ± 31.5	9.3	3.00 × 10^5^	This study
NrPinZ	(±)‐Syringaresinols (**4a/b**)	28.8 ± 6.4	794.4 ± 53.0	27.8	9.65× 10^5^	This study
NaPinZ	(±)‐Pinoresinols (**2a/b**)	97.6 ± 15.8	1842.0 ± 125.1	64.5	9.76 × 10^5^	This study
NaPinZ	(±)‐Medioresinols (**3a/b**)	24.5 ± 7.9	71.3 ± 7.6	2.5	1.02 ± 10^5^	This study
NaPinZ	(±)‐Syringaresinols (**4a/b**)	3.1 ± 0.9	596.7 ± 28.8	20.9	6.80 × 10^6^	This study
SlPinZ	(±)‐Pinoresinols (**2a/b**)	22.4 ± 2.5	135.9 ± 4.3	4.8	2.24 × 10^5^	This study
SlPinZ	(±)‐Medioresinols (**3a/b**)	13.3 ± 2.2	237.2 ± 7.5	8.3	6.26 × 10^5^	This study
SlPinZ	(±)‐Syringaresinols (**4a/b**)	11.2 ± 3.2	173.95 ± 9.6	6.1	5.46 × 10^5^	This study
AtPrR1	(+)‐Pinoresinol (**2a**)	1.6	0.6	0.02	1.3 × 10^4^	Nakatsubo et al. (2008)
AtPrR1	(−)‐Pinoresinol (**2b**)	7.3	3.9	0.14	1.9 × 10^4^	Nakatsubo et al. (2008)
PLR_Tp1	(+)‐Pinoresinol (**2a**)	320 ± 2.0	7.9 ± 0.0	0.56	1.8 × 10^3^	Hwang et al. (2020)
PLR_Tp1	(−)‐Pinoresinol (**2b**)	51 ± 9.0	5.8 ± 0.7	0.41	8.1 × 10^3^	Hwang et al. (2020)
PLR_Tp2^a^	(+)‐Pinoresinol (**2a**)	1.9 ± 0.0	33.0 ± 0.5	2.3	1.19 × 10^6^	Hwang et al. (2020)
PLR_Tp2^a^	(−)‐Pinoresinol (**2b**)	5.2 ± 0.1	12.7 ± 0.1	0.88	1.7 × 10^5^	Hwang et al. (2020)
IiPLR1	(±)‐Pinoresinols (**2a/b**)	65.4 ± 2.8	–	0.99	1.52 × 10^4^	Xiao et al. (2021)
*Native protein*						
PLR_Fi1^b^	(+)‐Pinoresinol (**2a**)	27 ± 1.5	4.5 ± 0.1	0.16	5820	Dinkova‐Kostova et al. (1996)
PLR_Fi2^b^	(+)‐Pinoresinol (**2a**)	23 ± 1.3	4.8 ± 0.1	0.17	7280	Dinkova‐Kostova et al. (1996)

Abbreviations: At: *Arabidopsis thaliana*; Fi: *Forsythia intermedia*; Ii: *Isatis indigotica*; Na: *Novosphingobium aromaticivorans*; Nr: *Novosphingobium rhizosphaerae*; Sl: *Sphingobium lignivorans*; Tp: *Thuja plicata*.

^a^ As PLR_Tp2 displayed severe substrate inhibition at high substrate concentrations, the Hill equation was used instead to obtain kinetic parameters, that is, *S*_0.5 instead_ of *K*_*m*_ and *k*_cat_/S_0.5_ instead of *k*_cat_/*K*_*m*_ (Hwang et al., 2020).

^b^ PLR_Fi1 and PLR_Fi2 native proteins were purified (~3000 fold) to homogeneity from ~20 kg *F. intermedia* stems over a 1 month period, hence the lower overall catalytic activity as compared to the recombinant proteins.

*Isatis* IiPLR1 was initially reported able to convert racemic pinoresinols (**2a/b**) into racemic secoisolariciresinols (**11a/b**) (Xiao et al., 2015). However, this data was not supported by either chiral column enantiomer composition analyses or measurements of optical activity of either substrates or products. A later publication (Chen et al., 2024) from the same laboratory reported that IiPLR1 reduced racemic pinoresinols (**2a/b**) into racemic lariciresinols (**6a/b**) with neither racemate apparently efficiently processed further. Curiously, these researchers provided no explanation as to why their earlier publication (Xiao et al., 2015) had racemic secoisolariciresinols (**11a/b**) as the final products of IiPLR1 catalysis. The actual biochemical function of IiPLR1 is, perhaps, somewhat uncertain.

In sum, plant and bacterial PR/PLR homologs can: (1) be enantiospecific (PLR1_Fi1, LuPLR2, and LuPLR1), generating essentially one secoisolariciresinol enantiomer; (2) convert both racemate isomers of pinoresinol (**2a/b**) into (near) racemic lariciresinols (**6a/b**) (NrPinZ, AtPrR1, and perhaps IiPLR1), that is, whereby only one of the two furanofuran rings is reduced; or (3) catalyze variations thereof (Tp_PLR1/Tp_PLR2).

Taken together, these findings underscored the need to better understand the catalytic processes that evolved in the various vascular plant and bacterial PLR/PLR homolog active sites, and how they differ, for example, as regards lack of enantiospecificity with NrPinZ, AtPrR1, and perhaps IiPLR1, *versus* that of plant PLRs with differing degrees of enantioselectivity. From an evolutionary standpoint, it is of particular interest that bacterial homologs reduce both pinoresinol enantiomers. This raised, in turn, the question as to whether during evolution they attained the ability to reduce both enantiomeric pinoresinol substructures in fern, gymnosperm and angiosperm lignins/lignans and their comparable homologous substructures. Such a capability would then have enabled the bacterial PLRs to biodegrade/recycle such homologous furanofuran substructures in order to produce simpler carbonaceous metabolites that could be utilized to support growth of successive generations of vascular and non‐vascular plants.

In the study herein, the substrate versatility and chiral outcomes of incubating NrPinZ with the aforementioned racemic furanofuran 8–8′ lignans, (±)‐syringaresinols (**4a/b**), (±)‐medioresinols (**3a/b**), and (±)‐ligballinols (**1a/b**) were determined, as well as for two other bacterial PLR homologs from *Novosphingobium aromaticivorans* F199 (Balkwill et al., 1997; Fredrickson et al., 1991) and *S. lignivorans* SYK‐6 (Fukuhara et al., 2013), respectively. This was to provide important insight into whether they were all able to catabolize racemic 8–8′ linked dimers that are found in different lignin type substructures or in 8–8′ linked lignans that are generally optically active.

Such an approach then allowed us to determine whether the bacterial PLRs studied herein were able to only process racemic pinoresinols (**2a/b**) found in gymnosperm and fern lignins, or were more substrate versatile being able to reduce the furofurano substructures (**2a/b**–**4a/b**) found in angiosperm lignins. The results so obtained then facilitated a second focus, via application of a combined approach of protein folding using Boltz‐2 and analysis of currently available PLR/PR homolog crystal structures, to explore why the NrPinZ, AtPrR1, and possibly IiPLR, bacterial PR homologs can reduce both enantiomeric forms of several 8–8′ linked furanofuran lignans efficiently, whereas the other plant PLRs can be enantiospecific, enantioselective, or variants thereof. Overall, our study provides important insights into the molecular basis of different stereochemical PLR/PR homolog outcomes in their respective enzymatic conversions. In turn, this provided new insights as regards hypothesized evolutionary/biological significance, as well as for enzyme engineering. In terms of the latter, knowledge gained of the binding pocket and catalytic properties will be essential for developing new biotechnological applications now and in the future.

## 2 RESULTS AND DISCUSSION

### 2.1 Bacterial and vascular plant PR/PLR sequence alignments and kinetic parameters

**FIGURE 3:**
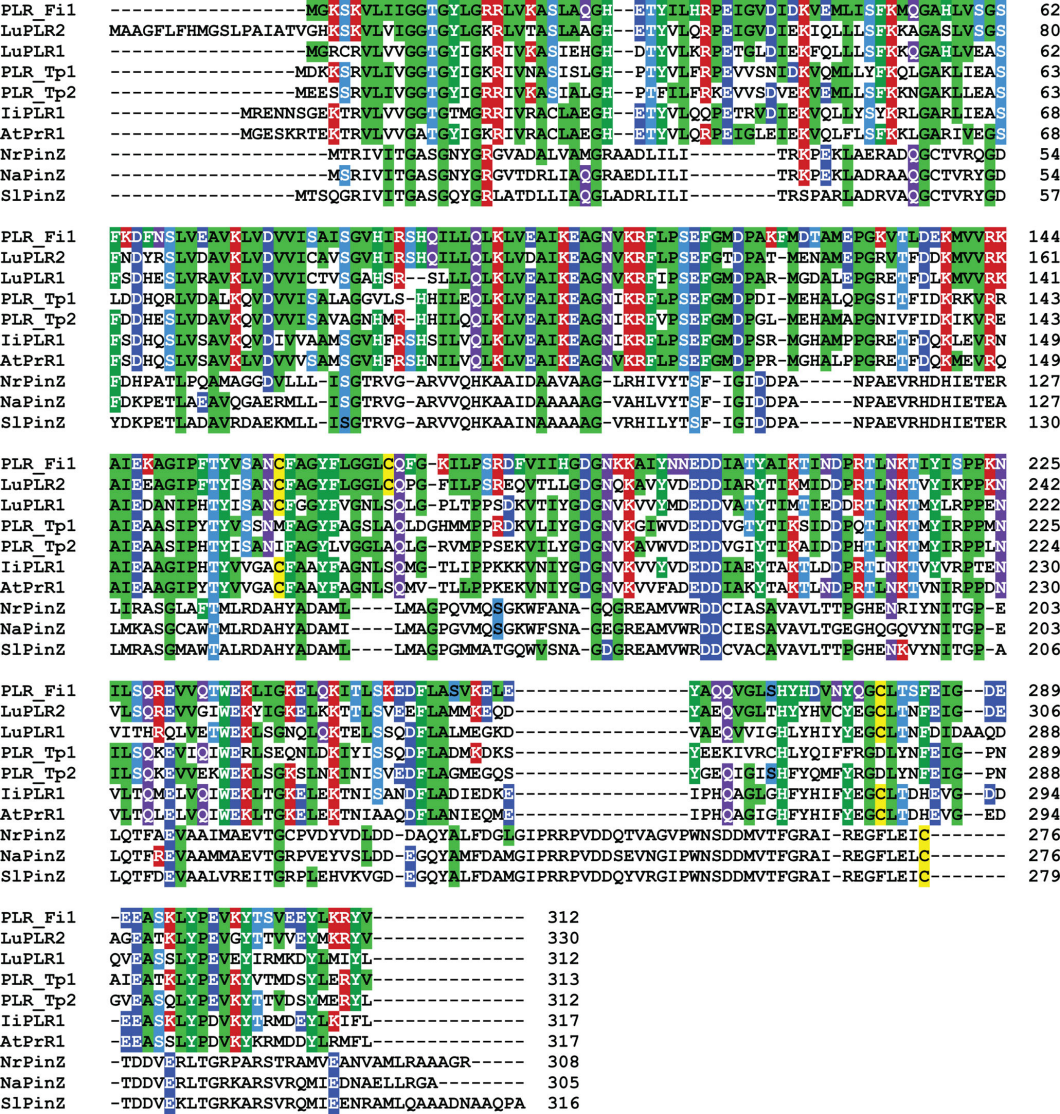
Structure‐based amino acid sequence alignment of plant PLRs (PLR_Fi1 from *Forsythia intermedia*, LuPLR1 and LuPLR2 from *Linum usitatissimum*, PLR_Tp1 and PLR_Tp2 from *Thuja plicata*, IiPLR1 from *Isatis indigotica*, AtPR1 from *Arabidopsis thaliana*), and bacterial PLR homologs NrPinZ, NaPinZ, and SlPinZ from *Novosphingobium rhizosphaerae, N. aromaticivorans*, and *Sphingobium lignivorans*, respectively. PLR_Fi1 and LuPLR2 are enantiospecific for (+)‐pinoresinol (**2a**); LuPLR1 is enantiospecific for (−)‐pinoresinol (**2b**). NrPinZ, AtPrR1, and possibly IiPLR1 convert racemic pinoresinols (**2a/b**) into (near) racemic lariciresinols (**6a/b**). PLR_Tp1 and PLR_Tp2 preferentially convert (−)‐pinoresinol (**2b**) and (+)‐pinoresinol (**2a**) into (+)‐secoisolariciresinol (**11a**) and (−)‐secoisolariciresinol (**11b**), respectively. However, PLR_Tp1 also slowly converts (+)‐pinoresinol (**2a**) into (+)‐lariciresinol (**6a**), and PLR_Tp2 slowly converts (−)‐pinoresinol (**2b**) into (−)‐lariciresinol (**6b**). NrPinZ, NaPinZ, and SlPinZ convert racemic pinoresinols (**2a/b**), medioresinols (**3a/b**), and syringaresinols (**4a/b**) into racemic **6–9a/b** (see Table 1).

Figure 3 shows amino acid sequence alignments of enantiospecific (+)‐PLRs, PLR_Fi1 and LuPLR2, (−)‐PLR LuPLR1, enantioselective PLRs PLR_Tp1 and PLR_Tp2, and PRs NrPinZ, AtPrR1, (and possibly IiPLR1) that convert (±)‐pinoresinols (**2a/b**) to the corresponding racemic lariciresinols (**6a/b**). Table 2 summarizes the percent identity comparisons between the enantiospecific (+)‐pinoresinol/lariciresinol reductase PLR_Fi1, other plant PLR/PR homologs, and the bacterial PinZ homologs. Of these, NrPinZ has very low identities to PLR_Fi1 and PLR_Tp2 (~18.7 and 19.8%, respectively), whereas PLR_Tp1 has 72.1% identity to PLR_Tp2. NaPinZ and SlPinZ are homologs to NrPinZ with 79.3 and 73.7% identities.

**TABLE 2:** Sequence comparison of bacterial PinZ and vascular plant PLR/PR homologs.

	Identity (%)								
	PLR_Fi1	LuPLR2	LuPLR1	PLR_Tp1	PLR_Tp2	IiPLR1	AtPrR1	NrPinZ	NaPinZ
LuPLR2	76.2								
LuPLR1	60.5	63.2							
PLR_Tp1	56.8	56.7	57.4						
PLR_Tp2	60.3	61.9	61.0	72.1					
IiPLR1	59.4	59.9	62.8	56.9	58.2				
AtPrR1	59.4	59.9	64.7	56.6	57.2	82.3			
NrPinZ	18.7	18.7	16.7	16.9	19.8	21.2	21.6		
NaPinZ	17.3	16.6	14.9	13.7	18.4	19.1	19.1	79.3	
SlPinZ	17.2	16.8	15.5	16.7	19.6	19.6	21.3	73.7	79.0

To determine whether NrPinZ, NaPinZ, and SlPinZ could process pinoresinol homologs, we synthesized racemic (±)‐ligballinols (**1a/b**), (±)‐medioresinols (**3a/b**), and (±)‐syringaresinols (**4a/b**), as well as their corresponding furan‐ring‐reduced derivatives as previously described (Hwang et al., 2020; Moinuddin et al., 2006). We then incubated each recombinant PR homolog with these potential substrates in the presence of NADPH and analyzed the resulting reactions using chiral‐phase HPLC separation; NADH, however, did not serve as a co‐factor (data not shown).

**FIGURE 4:**
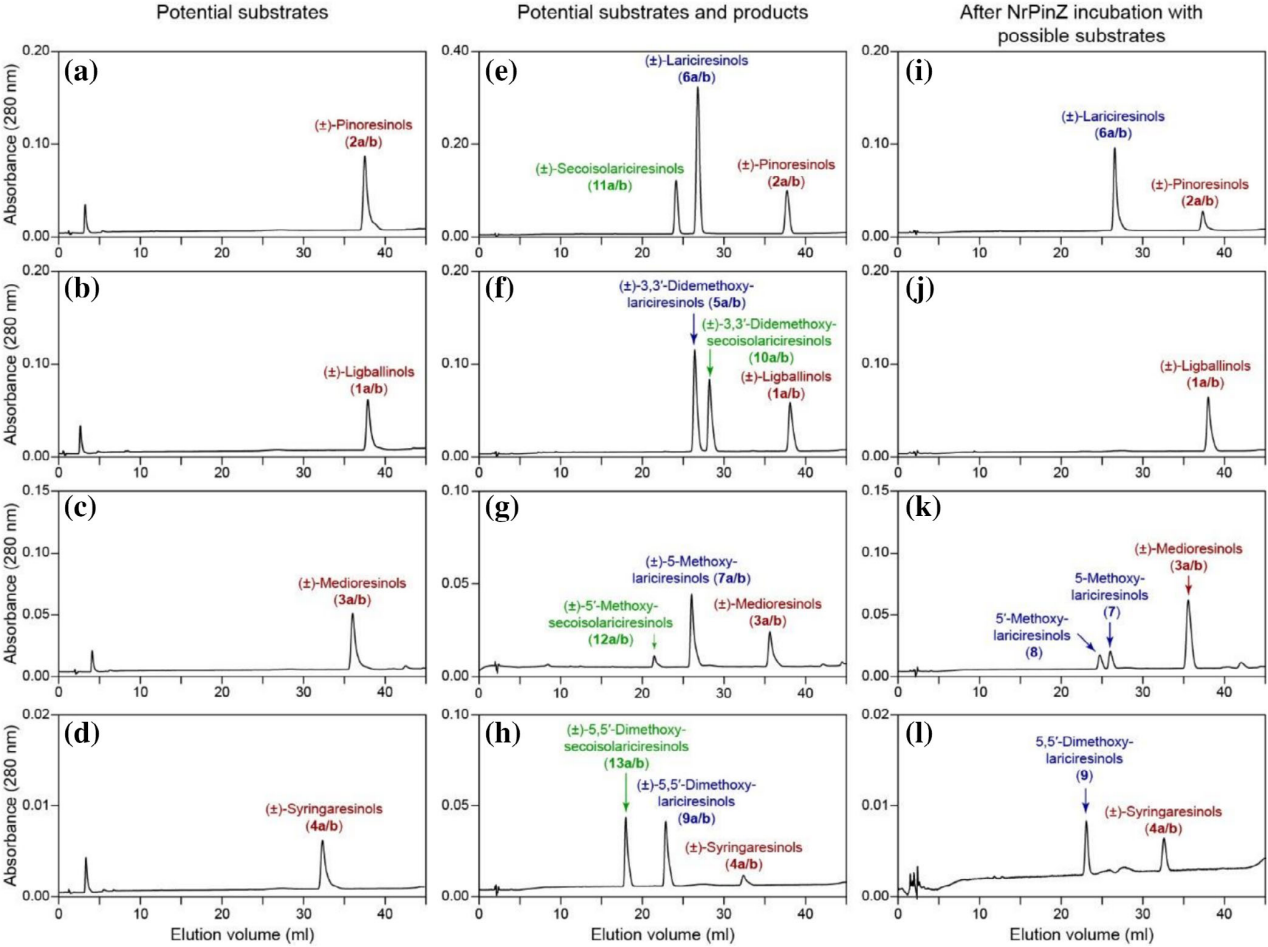
NrPinZ substrate versatility with (±)‐pinoresinols (**2a/b**), (±)‐ligballinols (**1a/b**), (±)‐medioresinols (**3a/b**), and (±)‐syringaresinols (**4a/b**). Reversed‐phase HPLC separations of NrPinZ potential 8–8′ substrates, (±)‐pinoresinols (**2a/b**), (±)‐ligballinols (**1a/b**), (±)‐medioresinols (**3a/b**), and (±)‐syringaresinols (**4a/b**) (a–d), and possible products (e–h) including those obtained from NrPinZ incubations for 10 min at 30°C (i–l).

Figure 4 shows HPLC reversed phase elution volumes of the authentic racemic furanofuran lignan potential substrates **1, 3** and **4** (Figure 4b–d), as well as their possible reduced racemic products **5**, **7**, **9**, **10**, **12** and **13** (Figure 4f–h). Focusing first on NrPinZ, its incubation results with the various substrates are shown in Figure 4j–l under the same reversed‐phase HPLC conditions: (±)‐ligballinols (**1a/b**) were not reduced (Figure 4j), whereas incubation with (±)‐medioresinols (**3a/b**) and (±)‐syringaresinols (**4a/b**) afforded the corresponding lariciresinol analogs **7–9** (Figure 4k,l). As medioresinol (**3**) is not symmetrical, a single furan ring reduction could form either of two products. Both such differentially reduced products **7** and **8** were observed after incubation of (±)‐medioresinols (**3a/b**) with NrPinZ (Figure 4k). (The analogous separations of the previously studied pinoresinol [**2a/b**] and lariciresinol [**6a/b**] standards, and NrPinZ catalyzed products are also included for comparison purposes [Figure 4a,e,i]).

**FIGURE 5:**
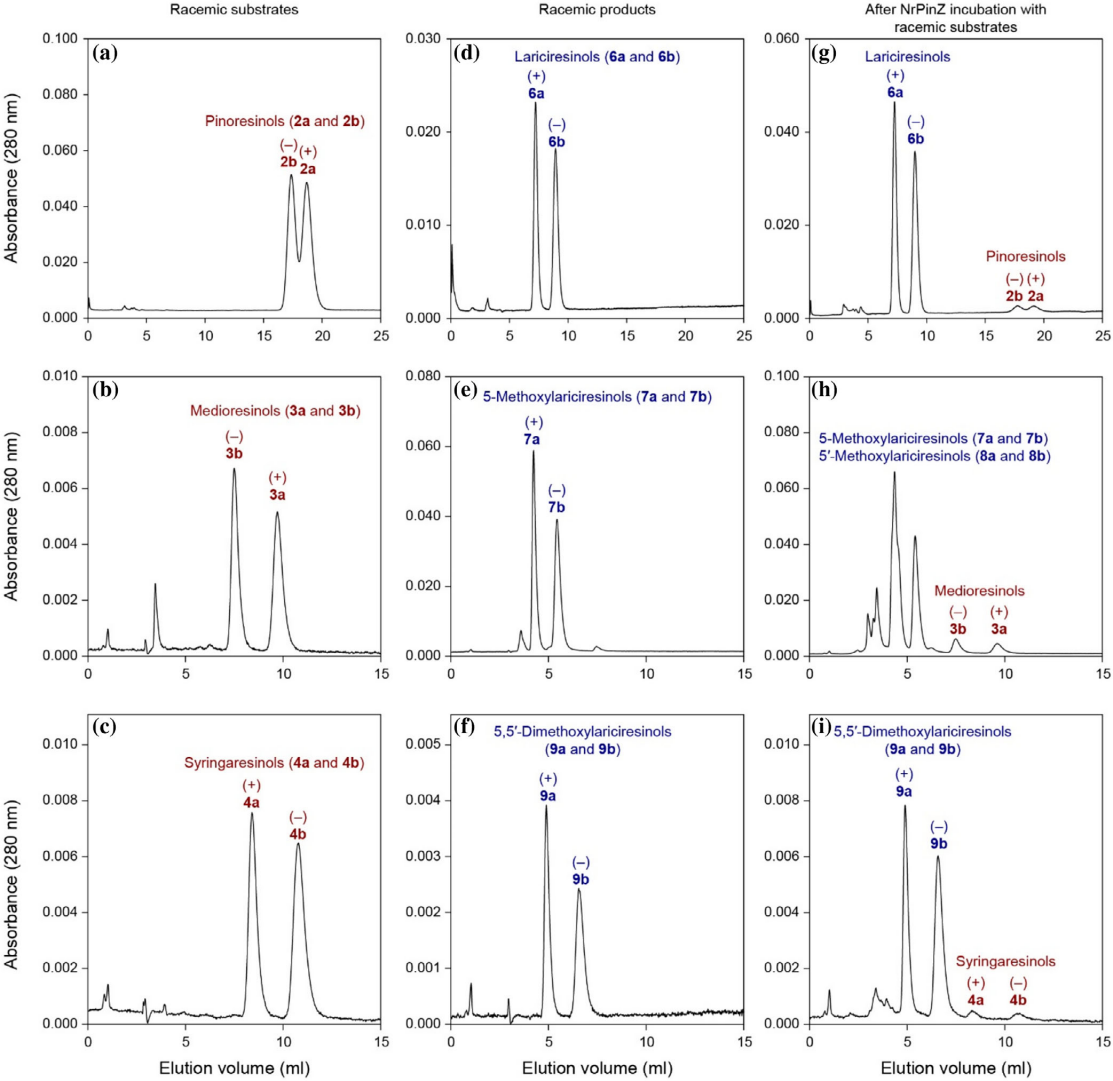
Chiral‐phase HPLC separations of NrPinZ substrates and products. Separations of (+) and (−)‐enantiomers of (a) pinoresinols (**2a** and **2b**), (d) lariciresinols (**6a** and **6b**), and results following incubation of NrPinZ with (g) (±)‐pinoresinols (**2a** and **2b**), using a Chiralcel OC column. Chiral analyses of (b) medioresinols (**3a** and **3b**), (c) syringaresinols (**4a** and **4b**), (e) 5‐methoxylariciresinols (**7a** and **7b**), and (f) 5,5′‐dimethoxylariciresinols (**9a** and **9b**) were conducted using a Chiralcel OD column, with results of NrPinZ incubation of substrates **3a/b** and **4a/b** shown in (h) and (I), respectively.

**FIGURE 6:**
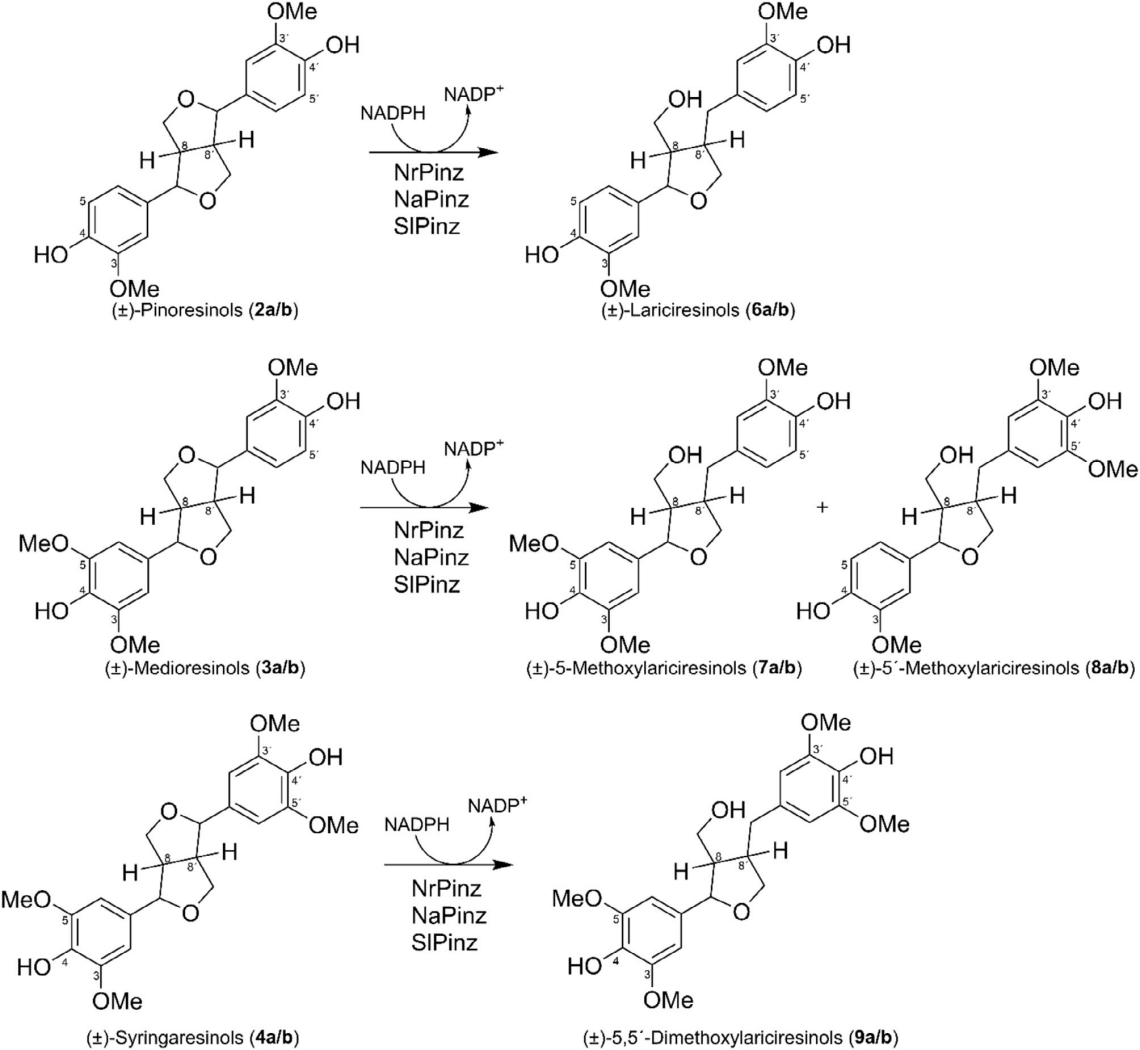
Reactions catalyzed by bacterial PLRs, NrPinZ, NaPinZ, and SlPinZ.

Chiral‐phase HPLC separations of authentic synthetic standards of (±)‐medioresinols (**3a/b**) and (±)‐syringaresinols (**4a/b**) (Figure 5b,c) were also developed, as were those of the corresponding potential enzymatic products above (Figure 5e,f), each giving well‐separated enantiomers. To assess NrPinZ potential substrate enantioselectivity with these substrates, the enzymatic products were next analyzed by chiral‐phase HPLC. Incubation with (±)‐medioresinols (**3a/b**) revealed a mixture of (+)‐ and (−)‐enantiomers of 5‐methoxylariciresinols (**7a/b**) and 5′‐methoxylariciresinols (**8a/b**) (Figure 5h), together with relatively small amounts of the remaining (±)‐medioresinol substrates (**3a/b**). Similarly, incubation with (±)‐syringaresinols (**4a/b**) generated the (+)‐ and (−)‐antipodes of 5,5′‐dimethoxylariciresinols (**9a/b**), together with residual amounts of (±)‐syringaresinols (**4a/b**) (Figure 5i). We therefore concluded that NrPinZ can also readily reduce both medioresinol (**3a/b**) and syringaresinol (**4a/b**) enantiomers but does not convert the H–H resinol ligballinols (**1a/b**). [Pinoresinols (**2a/b**), lariciresinols (**6a/b**), and NrPinZ catalyzed products are shown in Figures 5a,d,g and 6.]

In addition, both NaPinZ and SlPinZ bacterial PLR homologs were incubated with the same substrates and gave comparable results (Table 1 and Figure 6).

Steady‐state kinetic parameters of NrPinZ were next determined using both racemic (±)‐medioresinols (**3a/b**) and (±)‐syringaresinols (**4a/b**) (Table 1). Both racemic substrates were reduced equally well, as previously noted for (±)‐pinoresinols (**2a/b**) and (+)‐pinoresinol (**2a**) (88% e.e.) preparations, to afford the corresponding racemic (±)‐lariciresinol (**6a/b**) homologs. *K*_m_ determinations (30.9 ± 8.7 and 28.8 ± 6.4 μM) were also very similar to that previously determined for the (±)‐ and (+)‐pinoresinol substrates (**2a/b**), as was catalytic efficiency (*k*_cat_/*K*_m_; M^−1^ s^−1^) for all three different substrates (ranging from 3.00 to 9.65 × 10^5^). NaPinZ and SlPinZ gave comparable results. For NaPinZ with the same substrates (**2a/b–4a/b**), *K*_m_ determinations varied from 97.6 ± 15.8, 24.5 ± 7.9, and 3.1 ± 0.9 μM with corresponding *k*_cat_/*K*_m_ (M^−1^ s^−1^) values spanning 1.02 × 10^5^ to 6.8 × 10^6^. SlPinZ *K*_m_ determinations were relatively similar to NrPinZ 22.4 ± 2.5, 13.3 ± 2.2, and 11.2 ± 3.2 μM with *k*_cat_/*K*_m_ (M^−1^ s^−1^) spanning 2.24 to 6.26 × 10^5^.

Interestingly, the *N. rhizosphaerae* strain grows with pinoresinol (**2a/b**), with the latter catabolized in a five‐step enzymatic pathway as mentioned above (Allemann et al., 2025). In contrast, *N. aromaticivorans* and *S. lignivorans* have only the first pinoresinol (**2a/b**) catabolizing enzymatic pathway (NaPinZ and SlPinZ) step. This suggests that these two strains have alternate catabolism routes. Also noteworthy is NaPinZ's higher catalytic activity with syringaresinol (**4a/b**), relative to pinoresinol (**2a/b**) and medioresinol (**3a/b**); this may be a clue towards NaPinZ's native function.

It is perhaps instructive though that the overall catalytic efficiency (Table 1) of recombinant NrPinZ is *circa* 36 or 25‐fold higher than that estimated for AtPrR1 and possibly *circa* 31‐fold higher than IiPLR1 using racemic pinoresinol (**2a/b**) substrates. On the other hand, NrPinZ catalytic efficiency is *circa* 2.5‐fold less than PLR_Tp2 which preferentially utilizes (+)‐pinoresinol (**2a**). Nevertheless, NrPinZ is able to effectively catalyze reductions of (±)‐pinoresinols (**2a/b**), (±)‐medioresinols (**3a/b**), and (±)‐syringaresinols (**4a/b**), these also reflecting the different (C_9_) monomers present in the various lignin types.

### 2.2 Molecular modeling of NrPinZ and PLR_Tp2

**FIGURE 7:**
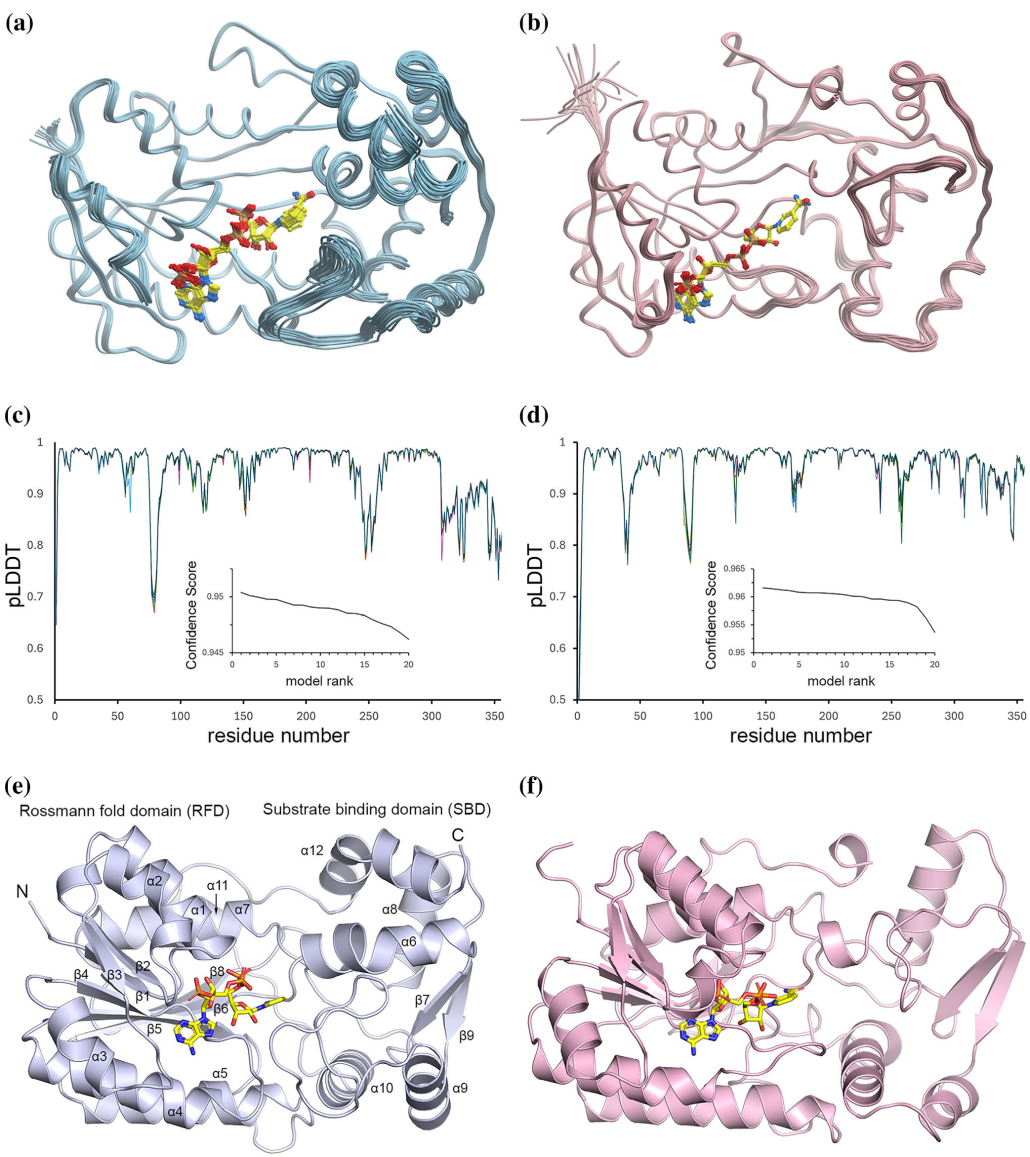
NrPinZ and PLR_Tp2 structural models. (a) The 20 Boltz‐2 NrPinZ diffusion models. The co‐folded NADPH molecules are shown with carbon atoms colored yellow, and oxygen, nitrogen and phosphorus atoms in standard coloring, red, blue and orange, respectively. In subsequent figures, only the carbon atom color will be specified. (b) The 20 Boltz‐2 PLR_Tp2 diffusion models, with the co‐folded NADPH molecules again shown as yellow sticks. (c) Plot of pLDDT as a function of residue number for the 20 NrPinZ models. The insert shows a plot of the Confidence Score (equivalent to 0.8 × pLDDT + 0.2 × ipTM) as a function of model number. (d) Plot of pLDDT as a function of residue number for the 20 PLR_Tp2 models. The insert shows a plot of the Confidence Score as a function of model number. (e) Top‐ranked Boltz‐2 predicted model for NrPinZ. The Rossmann fold domain (RFD) is on the left, and the substrate‐binding domain (SBD) is on the right. The NADPH is shown as yellow sticks. (f) Top‐ranked Boltz‐2 predicted model for PLR_Tp2, in the same orientation as “e.” The NADPH is shown as yellow sticks.

We compared and contrasted the 3D structures of relevant PR/PLR homologs, together with their putative cofactor and substrate binding models, to better understand their differing stereo‐specificities. While crystal structures have been reported for PLR_Tp1 (Min et al., 2003), IiPLR1 (Xiao et al., 2021), and AtPrR1 (Xiao et al., 2021), no structural data are available for NrPinZ or PLR_Tp2. To address this, we used the Boltz‐2 biomolecular structure prediction deep learning algorithm (Passaro et al., 2025) to generate the NADPH‐bound complexes of NrPinZ and PLR_Tp2, in which each enzyme and cofactor were co‐folded directly from their sequences (Figure 7a–d and Data S1 and S2). The top 20 ranked Boltz‐2 models for each enzyme complex (Figure 7a,b) were highly consistent, revealing a conserved architecture comprising a Rossmann fold domain (RFD) and a substrate‐binding domain (SBD). Superposition of the 20 Boltz‐2 models for each enzyme, aligned to their respective top‐ranked structures (Figure 7e,f), yielded mean root‐mean‐square deviations (*rmsd*s) in Cα positions of 0.42 Å for NrPinZ and 0.35 Å for PLR_Tp2, demonstrating strong convergence among the predicted conformations.

**FIGURE 8:**
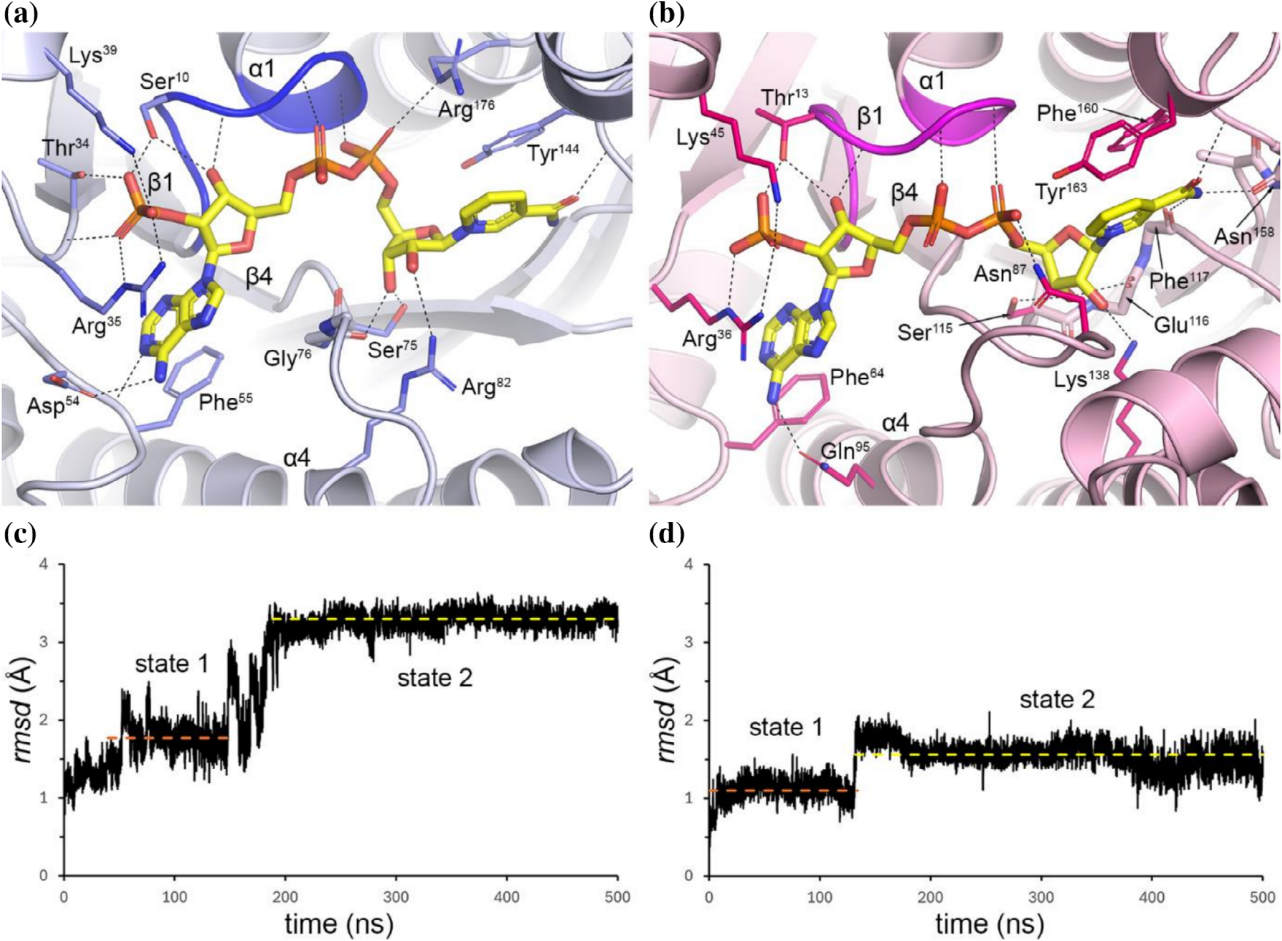
The NADPH binding site in the predicted models. (a) The top‐ranked Boltz‐2 model for NrPinZ (light blue ribbons) with the co‐folded NADPH cofactor shown as yellow sticks. The GxxGxxG Rossmann motif is colored blue. The residues which interact with the NADPH are shown as blue sticks, with hydrogen bonds shown as dashed black lines. (b) The top‐ranked Boltz‐2 model for PLR_Tp2 (light pink ribbons) with the co‐folded NADPH cofactor shown as yellow sticks. The GxxGxxG Rossmann motif is colored magenta. The residues which interact with the NADPH are shown as red sticks, with hydrogen bonds shown as dashed black lines. (c) Plot of the *rmsd* of the NADPH relative to frame 0 from the 500 ns MD simulation of NrPinZ‐NADPH. The first stable conformation (state 1) has an average *rmsd* of 1.8 Å (orange dashed line). The second stable conformation (state 2) has an average *rmsd* of 3.3 Å (yellow dashed line). (d) Plot of the *rmsd* of the NADPH relative to frame 0 from the 500 ns MD simulation of PLR_Tp2‐NADPH. The first stable conformation (state 1) has an average *rmsd* of 1.1 Å (orange dashed line). The second stable conformation (state 2) has an average *rmsd* of 1.6 Å (yellow dashed line).

The location and conformation of the co‐folded NADPH cofactor were then analyzed by taking the 20 superimposed models of each enzyme and calculating the average *rmsd* across all NADPH atoms. The resulting cofactor *rmsd*s were 0.4 Å for NrPinZ, and 0.2 Å for PLR_Tp2, and visual inspection confirmed that the NADPH was bound in an essentially identical conformation across all 20 models for each enzyme. From our previous study, it is known that plant PLRs have a conserved dinucleotide binding motif ^11^G*XX*G*XX*G^17^ located within the first β1‐α1‐β2 unit of the PLR_Tp1 RFD (Min et al., 2003). The corresponding motif in NrPinZ is ^8^G*XX*G*XX*G^14^. In the PLR_Tp2 NADPH‐binding pocket, the cofactor was modeled such that the Phe^160^ side chain forms an aromatic stacking interaction with the nicotinamide ring, while Lys^45^ forms a salt bridge with the 2′‐phosphate group (Figure 8b). The Phe^160^ residue is conserved among all the plant PLR/PR homologs but is replaced by tyrosine in bacterial enzymes. Likewise, Lys^45^ is conserved across both plant and bacterial PLR/PR homologs, with the exception of SlPinZ where the residue at the same position is Ser^39^ and is not considered functionally equivalent to lysine.

In the co‐folded NrPinZ and PLR_Tp2 complexes, the diphosphate group interacts primarily with the conserved G*XX*G*XX*G sequence motif, as expected, held by three hydrogen bonds between the phosphate oxygens and main chain amide nitrogen atoms (Figure 8a,b). The 2′‐phosphate group is anchored similarly in both enzymes, in a pocket lined by moderately conserved residues. In NrPinZ, these include Ser^10^, Thr^34^, Arg^35^ and Lys^39^ (Figure 8a), and in PLR_Tp2 the corresponding residues are Thr^13^, Arg^36^ and Lys^45^ (Figure 8b), consistent with the binding model developed for PLR_Tp1 (Min et al., 2003). In NrPinZ, the nicotinamide ring of the NADPH stacks face‐to‐face with Tyr^144^, with an additional stabilizing hydrogen bond formed between the main chain amide nitrogen of Tyr^144^ and the nicotinamide oxygen. The nicotinamide ribose interacts with Ser^75^ and Gly^76^ from the C‐terminus of strand β4, and with Arg^82^ from the N‐terminus of helix α4 (Figure 8a). In PLR_Tp2, the nicotinamide is anchored by two aromatic interactions with Phe^160^ and Tyr^163^, and by hydrogen bonds involving main chain atoms of Phe^117^, Asn^158^ and Phe^160^ (Figure 8b). The nicotinamide ribose moiety forms hydrogen bonds with Ser^115^, Glu^116^ and Lys^138^.

The relative stability of NADPH binding in both the NrPinZ and PLR_Tp2 Boltz‐2 top‐ranked models was further assessed by 500 ns molecular dynamics (MD) simulations performed in triplicate. In the NrPinZ‐NADPH simulation, the average *rmsd* of the NADPH relative to the starting model was 2.7 Å, and a plot of the *rmsd* *versus* time (Figure 8c) revealed two distinct conformational populations of the cofactor. Visual inspection of the MD trajectory showed that although the adenosine ribose and diphosphate moieties remained structurally stable throughout the simulation, variance in *rmsd* rose primarily from changes in the orientation of the 2′‐phosphate and the adenine groups, producing conformational state 1 (*rmsd* ~ 1.8 Å) between 50 and 150 ns. After ~200 ns, additional displacement of the nicotinamide riboside led to conformational state 2 (*rmsd* ~3.3 Å). Although two conformational states are also observed in the PLR_Tp2 simulation (Figure 8d), the average *rmsd*s of the NADPH cofactor relative to the starting model were significantly lower (~1.1 and ~1.6 Å in states 1 and 2, respectively), and the nicotinamide riboside does not display a large shift in orientation. These results suggest that the nicotinamide riboside is more firmly anchored in PLR_Tp2 than in the bacterial enzyme.

**FIGURE 9:**
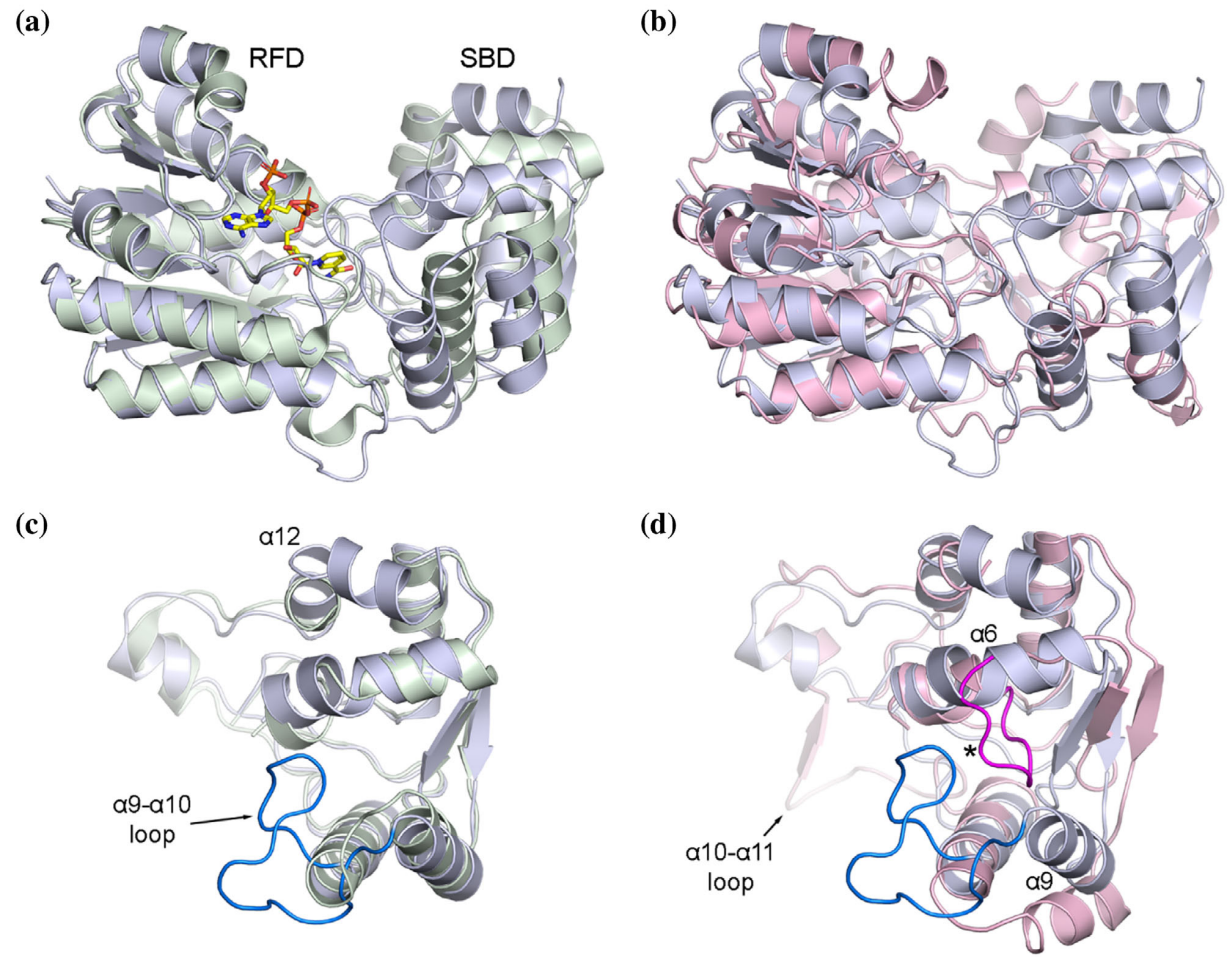
Comparison of reductase structures. (a) Superposition of the top‐ranked NrPinZ model (light blue) onto TMR (pale green), based on alignment of the respective Rossmann fold domains. The substrate‐binding domains can be superimposed with a *rmsd* of 1.6 Å and are offset by a rotation and translation of 3.5° and 1.7 Å, respectively. The NADP bound to TMR is shown as yellow sticks. (b) Superposition of NrPinZ (light blue) onto PLR_Tp2 (pink) based on alignment of the respective Rossmann fold domains. (c) Superposition of the NrPinZ (light blue) and TMR (pale green) SBDs. The α9‐α10 loop extension in NrPinZ is shown in blue. (d) Superposition of the NrPinZ (light blue) and PLR_Tp2 (pink) SBDs. The α9‐α10 loop extension in NrPinZ is shown in blue, and the loop which shortens the α6 helix in PLR_Tp2 is colored red and indicated by the asterisk. The α10‐α11 loop extension and displacement of helix α9 in PLR_Tp2 is evident.

Using the top‐ranked NrPinZ Boltz‐2 model (Figure 7e), Dali searches (Holm et al., 2023) of the Protein Data Bank (PDB) revealed strong structural matches to several enzymes, including quinone oxidoreductase (QOR2; 2zcv), triphenylmethane reductase (TMR; 2vrb), an epimerase (3e48), a monooxygenase (5f5n), a polyketide reductase (5l45) and NADH–ubiquinone oxidoreductase chain 3 (8ca3) (Table 3). AtPrR1, PLR_Tp1, and IiPLR1 also gave strong structural matches, albeit with slightly lower but still highly significant Z‐scores. Superpositions of the top‐ranked NrPinZ Boltz‐2 model onto QOR2 (not shown) and TMR (Figure 9a), based on alignment of the RFD only, yielded *rmsd*s of 1.4 and 1.2 Å, respectively. A corresponding Dali search using the top‐ranked PLR_Tp2 Boltz‐2 model (Figure 7f) similarly identified close structural matches with AtPrR1 (7cse), PLR_Tp1 (1qyd), and IiPLR1 (7cs3), along with phenylcoumaran benzylic ether reductase (PCBER; 1qyc) (Min et al., 2003), an isoflavone reductase from *Medicago sativa* (2gas), and eugenol synthase 1 from *Ocimum basilicum* (2qw8) (Table 4).

**TABLE 3:** Selected DALI search results for the top‐ranked NrPinZ Boltz‐2 model.

DALI order	Enzyme	Preferred nucleotide	PDB	Z‐score	*rmsd* (Å)	Residues matched
1	Quinone oxidoreductase, QOR2	NADP	2zcv	37.4	1.4	283
2	Triphenylmethane reductase (TMR)	NADP	2vrb	36.2	1.7	282
8	Epimerase	unknown	3e48	34.9	1.5	276
11	Monooxygenase	NAD	5f5n	30.1	2.2	277
13	SIMC7 polyketide reductase	NADP	5 l45	29.7	2.9	277
20	NADH–ubiquinone oxidoreductase chain 3	NAD	8ca3	27.8	2.5	284
21	NMRA‐like family domain	NADP	3dxf	27.7	2.6	276
176	Pinoresinol reductase, AtPrR1	NADP	7csd	24.5	3.2	276
205	Pinoresinol‐lariciresinol reductase, PLR_Tp1	NADP	1qyd	24.1	3.2	276
210	Eugenol synthase 1	NADP	2r6j	23.7	3.2	274
220	Isoflavone reductase	NADP	2gas	23.6	3.5	272
224	Phenylcoumaran benzylic ether reductase (PCBER)	NADP	1qyc	23.5	3.4	272
229	Pinoresinol‐lariciresinol reductase, IiPLR1	NADP	7cs3	23.4	3.3	269

**TABLE 4:** Selected DALI search results for the top‐ranked PLR_Tp2 Boltz‐2 model.

DALI order	Enzyme	Preferred nucleotide	PDB	Z‐score	*rmsd* (Å)	Residues matched
1	Pinoresinol reductase, AtPrR1	NADP	7cse	45.9	1.0	303
26	Pinoresinol‐lariciresinol reductase, PLR_Tp1	NADP	1qyd	45.0	1.1	306
34	Phenylcoumaran benzylic ether reductase (PCBER)	NADP	1qyc	43.2	1.3	301
40	Pinoresinol‐lariciresinol reductase, IiPLR1	NADP	7cs3	41.6	1.2	290
50	Isoflavone reductase	NADP	2gas	40.5	1.5	298
71	Eugenol synthase 1	NADP	2qw8	39.8	1.5	292
99	Leucoanthocyanidin reductase 1	NADP	3i6i	38.5	1.6	293
105	Monooxygenase	NAD	5f5n	25.8	2.9	266
109	NADH–ubiquinone oxidoreductase chain 3	NAD	8bee	25.6	3.0	273
156	Triphenylmethane reductase (TMR)	NADP	2vrb	24.5	3.1	267
170	Quinone oxidoreductase, QOR2	NADP	2zcv	24.4	2.8	265

Superposition of the NrPinZ and PLR_Tp2 Boltz‐2 models revealed the structural similarity of the RFDs (*rmsd* ~2.0 Å) and greater structural variance in the SBDs (Figure 9b). Similar superpositions of NrPinZ with AtPrR1, IiPLR1, and PLR_Tp1, based on the RFD only, gave *rmsd*s of 2.1, 2.4, and 2.2 Å, respectively, whereas superpositions of the top‐ranked PLR_Tp2 models on AtPrR1, IiPLR1, and PLR_Tp1 yielded *rmsd*s of 0.83, 0.78, and 1.0 Å, respectively (data not shown). The RFDs and SBDs are essentially identical in these four plant enzymes and the spatial disposition of the SBD relative to the RFD is conserved across all. Given the high degree of structural similarity in the RFD between NrPinZ and TMR (Figure 9a), the presence of a bound NADPH in TMR provided an independent means to validate the co‐folded NrPinZ‐NADPH complex. Superposition of the TMR structure onto the 20 Boltz‐2 NrPinZ models, based on the RFD, followed by calculation of the NADPH *rmsd*s, gave an average displacement of 1.2 Å. Visual inspection confirmed that the NrPinZ cofactors closely overlapped the position of the NADPH in TMR. To further verify the cofactor conformation in the co‐folded PLR_Tp2‐NADPH complexes, the 20 Boltz‐2 structures were compared to the NADP^+^‐bound structure of AtPrR1 (PDB code 7csa). The calculated average NADPH displacement was 1.4 Å, comparable to the *rmsd* obtained from the MD simulation, supporting the stability and accuracy of the co‐folded cofactor conformations.

The SBDs of NrPinZ and TMR, although topologically similar to the plant PR/PLR homologs, display notable structural diversity, both in overall domain positioning relative to the RFD and in more nuanced differences in some loops and secondary structure elements. The TMR SBD closely resembles the equivalent domain in NrPinZ and superimposes with a *rmsd* of 1.6 Å (Figure 9a). The primary structural differences occur in the loop between helices α9 and α10 (Figure 9c), where in NrPinZ there is an insertion of 12 residues (Pro^245^‐Trp^256^), and at the C‐terminus where helix α12 is elongated by nine residues and kinked by almost 90° in its midpoint. In contrast, the SBDs of AtPrR1, IiPLR1, PLR_Tp1, and PLR_Tp2 diverge somewhat in structure relative to NrPinZ and TMR. The major structural difference is in helix α6, which in NrPinZ comprises ~13 residues and leads directly into strand β7, whereas in the plant enzymes, this helix is only six residues long and is followed by a nine‐residue loop projecting roughly perpendicular to the helix axis. This extended loop results in displacement of helix α9 by approximately 6–8 Å from its position in NrPinZ (Figure 9d). Finally, the loop following helix α10 is about six residues longer in PLR_Tp1 and PLR_Tp2 and incorporates an additional short β‐strand running parallel to strand β8 before leading into helix α11.

**FIGURE 10:**
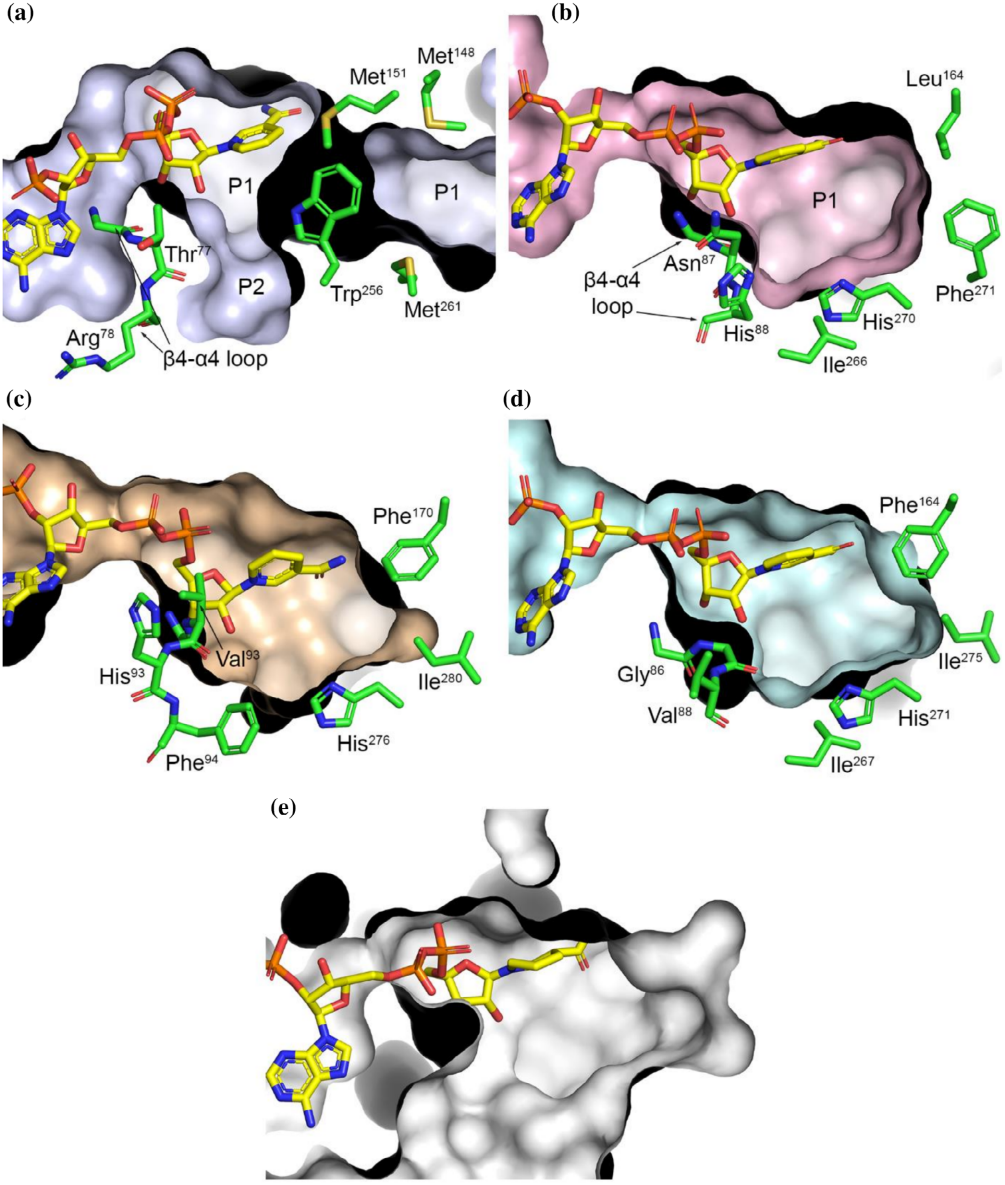
The substrate binding pocket (SBP). (a) Molecular surface representation of the NrPinZ SBP showing two distinct sub‐pockets, P1 and P2. The side chains of Met^151^ and Trp^256^ form a bridge over the P1 pocket. (b) Surface representation of the PLR_Tp2 SBP. The Asn^87^ and His^88^ side chains block formation of a P2 pocket. (c) Surface representation of the AtPrR1 SBP (PDB code 7csa). (d) Surface representation of the PLR_Tp1 SBP (PDB code 1qyd) (e) Surface representation of the IiPLR1 SBP (PDB code 7cs3). In panels “a, b, c and e”, bound NADPH is shown as yellow sticks. For panel “d”, where NADPH was not in the crystal structure of PLR_Tp1, the cofactor location in the homolog PLR_Tp2 is shown. In panels “a, b, c and d”, selected side chains are shown as green sticks.

The substrate‐binding pocket within the SBD differs markedly between the bacterial and plant PLRs. In NrPinZ, the pocket is divided into two regions, a large cavity (P1) extending outward from the nicotinamide and a smaller pocket (P2) at approximately 90° relative to P1 (Figure 10a). The α9‐α10 loop extension folds across the top of the binding site, with the side chain of Trp^256^ forming a hydrophobic bridge with Met^151^ over the P1 pocket. In contrast, PLR_Tp2 lacks a distinct P2 pocket. A difference in the structure of the β4‐α4 loop, resulting from the insertion of an additional residue in the plant enzymes, effectively prevents P2 pocket formation (Figure 10b). The intrusion of the Leu^164^ and Phe^271^ side chains into the P1 pocket alters the shape of the pocket, making it significantly shorter relative to NrPinZ. Molecular surface calculations of AtPrR1 (Figure 10c) and PLR_Tp1 (Figure 10d) similarly reveal a single‐pocket architecture, with the β4‐α4 loop again sealing off any potential P2 cavity. We also modeled the IiPLR1 binding pocket, though interpretation remains tentative given the ongoing uncertainty regarding the enzyme's physiological substrates and products. Consequently, no definitive functional conclusions can be drawn at present. Nonetheless, the substrate‐free IiPLR1–NADP^+^ structure (PDB 7csa) appears to possess a single, comparatively broader pocket (Figure 10e) than those observed in other plant PLRs.

### 2.3 Molecular docking of substrates into NrPinZ and PLR_Tp2 NADPH complexes

**FIGURE 11:**
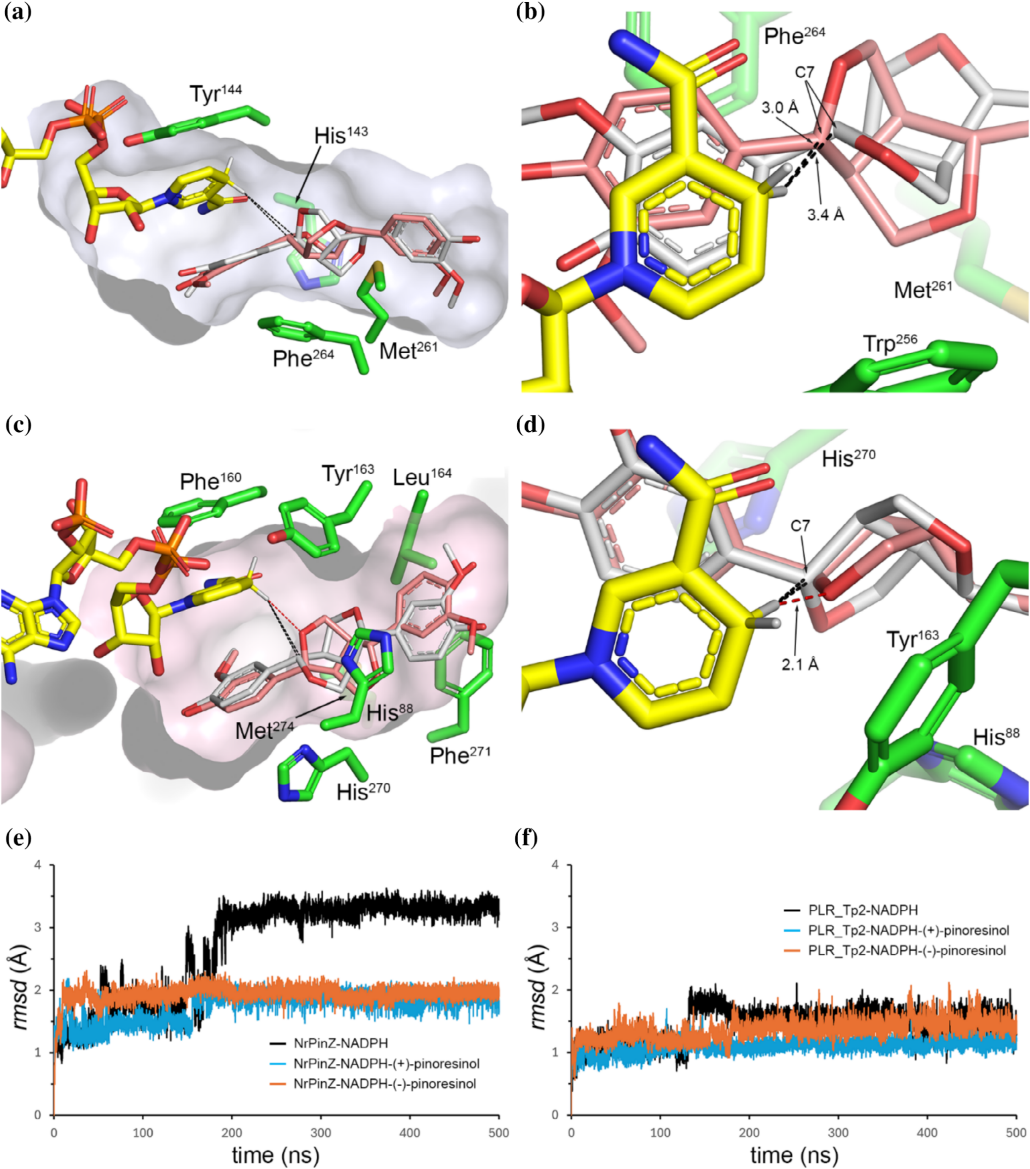
Molecular docking. (a) Molecular docking poses for (+)‐pinoresinol (**2a**—thin gray sticks) and (−)‐pinoresinol (**2b**—thin pink sticks) in the NrPinZ receptor. The distance from the transferable proton of the nicotinamide and the C7 atom of both enantiomers is indicated by black dashed lines. The Trp^256^ side chain is not shown for clarity. (b) View of the two NrPinZ docked complexes looking down onto the nicotinamide. The proton‐C7 distances are indicated. (c) Poses for (+)‐ and (−)‐pinoresinol (**2a** and **2b**—thin gray and pink sticks, respectively) in the PLR_Tp2 receptor, with the distances from the nicotinamide transferable proton to the substrate C7 indicated as black dashed lines. A steric clash between the proton and the furan oxygen in the (−)‐pinoresinol complex is indicated by a red dashed line. (d) View of the two PLR_Tp2 docked complexes looking down onto the nicotinamide. The proton‐*O* distance is indicated. The distances between the proton and the C7 atoms are not shown for clarity (2.9 Å for (+)‐pinoresinol and 3.4 Å for (−)‐pinoresinol). In panels “a, b, c and d”, bound NADPH is shown as yellow sticks, and selected side chains as green sticks. (e) Plot of the *rmsd* of the NADPH relative to frame 0 from the 500 ns MD simulation for NrPinZ‐NADPH (black trace), NrPinZ‐NADPH‐(+)‐pinoresinol (cyan trace) and NrPinZ‐NADPH‐(−)‐pinoresinol (orange trace). (f) Plot of the *rmsd* of the NADPH relative to frame 0 from the 500 ns MD simulation for PLR_Tp2‐NADPH (black trace), PLR_Tp2‐NADPH‐(+)‐pinoresinol (cyan trace) and PLR_Tp2‐NADPH‐(−)‐pinoresinol (orange trace).

To gain some insights into the stereoselectivity of the bacterial and plant PLRs, the top‐ranked NrPinZ‐NADPH and PLR_Tp2‐NADPH models were used as molecular receptors for docking of both enantiomers of pinoresinol (**2a/b**; Figure 11a,b), medioresinol (**3a/b**), and syringaresinol (**4a/b**) using ICM‐Pro (Abagyan & Totrov, 1994) (Table 5). In the NrPinZ docking calculations, the majority of the poses positioned both enantiomers within the P1 pocket (Figure 11a), with the C7 atom located 3.0–3.4 Å from the transferable proton on the nicotinamide ring (Figure 11b), close enough to permit efficient hydride transfer for either isomer. Substrate binding is primarily driven by π‐π stacking of the proximal *o*‐methoxyphenol ring against the nicotinamide face opposite Tyr^144^, together with enclosure of the fused furanofuran rings in a hydrophobic tunnel delineated by the side chains of His^143^, Met^261^, Phe^264^ (Figure 11a) and Trp^256^ (Figure 11b). The distal *o*‐methoxyphenol ring makes no contacts with the enzyme in the widened P1 pocket beyond the Met^151^‐Trp^256^ bridge. Although there are additional hydrogen bonding interactions between the phenolate oxygen and Arg^82^ (data not shown), the overall binding stability for both enantiomers is dominated by the extensive aromatic and hydrophobic interactions within the P1 pocket.

**TABLE 5:** ICM‐Pro docking results^a^.

Complex	Docking score	mfScore	Hbond	Hphob	VwInt	Eintl
			kJ/mol	kJ/mol	kJ/mol	kJ/mol
NrPinZ‐NADPH‐(+)‐pinoresinol	−18.1	−157.8	−1.6	−7.5	−34.9	4.4
NrPinZ‐NADPH‐(−)‐pinoresinol	−16.4	−135.0	−3.4	−8.1	−30.3	3.7
NrPinZ‐NADPH‐(+)‐syringaresinol	−16.3	−137.8	−3.2	−9.3	−40.5	6.7
NrPinZ‐NADPH‐(−)‐syringaresinol	−12.7	−120.6	−3.3	−9.3	−33.8	7.9
NrPinZ‐NADPH‐(+)‐medioresinol	−16.9	−125.8	−4.3	−8.5	−38.6	4.1
NrPinZ‐NADPH‐(−)‐medioresinol	−19.8	−144.4	−3.5	−8.9	−32.7	3.0
PLR_Tp2‐NADPH‐(+)‐pinoresinol	−16.1	−79.3	−5.7	−7.5	−22.5	7.7
PLR_Tp2‐NADPH‐(−)‐pinoresinol	−17.6	−84.4	−5.7	−7.4	−25.2	8.6
PLR_Tp2‐NADPH‐(+)‐syringaresinol	−8.8	−84.1	−3.3	−8.2	−26.5	9.1
PLR_Tp2‐NADPH‐(−)‐syringaresinol	−5.2	−80.3	−2.9	−8.5	−21.2	14.6
PLR_Tp2‐NADPH‐(+)‐medioresinol	−15.8	−73.9	−5.4	−8.1	−21.5	9.1
PLR_Tp2‐NADPH‐(−)‐medioresinol	−9.8	−76.6	−5.2	−8.2	−18.2	10.8

Abbreviations: Eintl, internal conformation energy of the ligand; Hbond, hydrogen bond energy; Hphob, hydrophobic energy in exposing a surface to water; VwInt, van der Waals interaction energy.

^a^ Energy terms from ICM‐Pro are as follows: Docking score, a dimensionless value calculated from the energy terms; mfScore, potential of mean force score. This is an independent score of the strength of ligand‐receptor interaction. This value represents the energy difference between the relaxed (unbound) ligand and its conformation in the docked (bound) state. The larger the value, the more strained the conformation.

In contrast, the smaller pocket in PLR_Tp2 restricts the orientation of the substrates in the P1 pocket. The proximal *o*‐methoxyphenol is stacked against the same face of the nicotinamide as in NrPinZ, with the furanofuran rings tightly wedged between the side chains of His^88^, Tyr^163^, His^270^ and Met^274^ (Figure 11c). Unlike NrPinZ, however, the distal *o*‐methoxyphenol ring is anchored by hydrophobic and aromatic interactions with His^88^, Leu^164^, and Phe^271^, resulting in a more conformationally constrained substrate orientation within the pocket. The docking results suggest that the (+)‐enantiomers adopt a geometry consistent with productive hydride transfer (~2.8 Å between the transferable proton on the nicotinamide and the C7), whereas in the (−)‐enantiomers, the proximal furanyl oxygen points directly towards the nicotinamide, potentially introducing steric hindrance to hydride transfer (Figure 11d). Although comparison of the reported (±)‐pinoresinol crystal structures of AtPrR1 and IiPLR1 could, in principle, help assess the validity of the docking results, inspection of the available crystal structures (PDB codes 7csb, 7csc, 7cs4, and 7cs5) reveals that the furanofuran rings of the bound substrates are severely distorted from ideal geometry and, in some cases, poorly fitted to the electron density. As such, these structures cannot be used for meaningful comparison.

To assess the stability of the ternary complexes, triplicate 500 ns MD simulations were performed for the NrPinZ‐NADPH and PLR_Tp2‐NADPH complexes of (+)‐ and (−)‐pinoresinols (**2a** and **2b**). In NrPinZ, the simulation revealed substantial stabilization of NADPH in the presence of either enantiomer (Figure 11e). Visual inspection of the trajectories showed that the nicotinamide riboside moiety, which was previously dynamic in the binary complex, became conformationally constrained within the P1 pocket, sandwiched between Tyr^144^ and the *o*‐methoxyphenol ring of the substrate. In PLR_Tp2, NADPH was already more stable relative to NrPinZ, and only a modest reduction of the *rmsd*s was observed upon MD simulation with either enantiomer of pinoresinol (Figure 11f), consistent with the more compact and rigid active‐site architecture of the plant enzyme. Whether the presence of a P2 pocket in NrPinZ, in contrast to its absence in the plant enzymes, has a specific functional role remains unclear based on the current models. The enlarged pocket volume in NrPinZ may account for the greater mobility of the free nicotinamide moiety observed in the binary NrPinZ‐NADPH simulations. In the ternary NrPinZ complexes, although the P2 pocket is not directly involved in substrate binding, the additional space adjacent to the nicotinamide may permit transient substrate excursions into this region, potentially facilitating the adoption of productive conformations by either enantiomer. In contrast, the absence of this extra volume in PLR_Tp2 likely restricts the available conformational space, resulting in the sterically perturbed docking poses observed for the (−)‐pinoresinol complex.

These molecular modeling results are consistent with the enzymatic activities observed *in vitro*, and provide a plausible structural basis for the observed ability of NrPinZ to convert all (±)‐pinoresinols (**2a/b**), (±)‐medioresinols (**3a/b**), and (±)‐syringaresinols (**4a/b**) into racemic lariciresinols (**6a/b**) and analogs (**7–9a/b**). Moreover, since PLR_Tp2 readily reduces (+)‐pinoresinol (**2a**) (*S*_0.5_ 1.9 μM, *k*_cat_/*S*_0.5_ 1.19 × 10^6^ M^−1^ s^−1^) to afford (−)‐secoisolariciresinol (**11b**), the reaction with (−)‐pinoresinol (**2b**) [to only give (−)‐lariciresinol (**6b**)] has lower overall catalytic efficiency (*S*_0.5_ 5.2 μM and *k*_cat_/S_0.5_ 1. 7 × 10^5^ M^−1^ s^−1^). This reduction in activity is consistent with the smaller, more conformationally restricted binding pocket of PLR_Tp2 and the apparent inability of the (−)‐pinoresinol enantiomer to adopt a geometry favorable for productive hydride transfer.

## 3 CONCLUSIONS

The abilities of NrPinZ, NaPinZ, and SlPinZ to catabolize the different racemic 8–8′ furanofuran lignans, (±)‐pinoresinols (**2a/b**), medioresinols (**3a/b**), and syringaresinols (**4a/b**) into racemic lariciresinols (**6a/b**) and its analogs (**7–9a/b**) are potentially biologically very significant. 8–8′‐Linked lignin sub‐structures or 8–8′ lignan structures are considered formed via coupling of coniferyl, 5‐hydroxyconiferyl, and sinapyl alcohols, respectively. In lignins, coniferyl alcohol polymerization in ferns and gymnosperms results in the formation of so‐called guaiacyl (G) lignins, and those from both coniferyl and sinapyl alcohols are the corresponding guaiacyl/syringyl (G/S) lignins in angiosperms.

The ability of *N. rhizosphaerae* to grow on (+)‐pinoresinol (**2a**) and (−)‐syringaresinol (**4b**), as single carbon sources, and in catabolizing pinoresinol (**2a**) into coniferyl aldehyde and vanillin may have broader ramifications, that is, in the sense that a similar catabolism process can be anticipated for syringaresinol (**4**) and medioresinol (**3**). Isolation of *N. rhizosphaerae* from a Yellowstone hot spring may also suggest that, in a geographical location where both woody and non‐woody gymnosperms and angiosperms are present, *N. rhizosphaerae* has acquired the ability to catabolize all such lignans as well as the corresponding sub‐structure entities present in both G and G/S lignins. This is also possibly the case for the bacterial NaPinZ and SlPinZ homologs. However, from our previous studies of plant PLR substrate versatility, we know that plant PLRs are unable to metabolize furanofuran lignans lacking free phenolic groups at C4 and C4′ such as (+)‐sesamin (Hwang et al., 2020). Although not explored in this study, it may be that the *N. rhizosphaerae* and the other bacterial lignin catabolism process may first require etherified 8–8′ lignin sub‐structures to undergo catabolism to free either one or two free phenolic groups at C4 and C4′.

The Boltz‐2 modeling was also very instructive. The binding and catabolism of the bound 8–8′ furanofuran lignans in the elongated NrPinZ P1 pocket allow for both racemate forms to be catabolized. Conversely, the smaller and more constrained P1 pocket in PLR_Tp2 may enforce a binding geometry that preferentially positions (+)‐pinoresinol (**2a**) for efficient hydride transfer and subsequent downstream metabolism.

## 4 MATERIALS AND METHODS

### 4.1 Substrate and product syntheses

Racemic (±)‐pinoresinols (**2a/b**), (±)‐lariciresinols (**6a/b**), and (±)‐secoisolariciresinols (**11a/b**) were synthesized as reported (Moinuddin et al., 2006), whereas (±)‐ligballinols (**1a/b**), (±)‐medioresinols (**3a/b**), (±)‐syringaresinols (**4a/b**), (±)‐3,3′‐didemethoxylariciresinols (**5a/b**), (±)‐5‐methoxylariciresinols (**7a/b**), (±)‐5′‐methoxylariciresinols (**8a/b**), (±)‐5,5′‐dimethoxylariciresinols (**9a/b**), (±)‐3,3′‐didemethoxysecoisolariciresinols (**10a/b**), (±)‐5′‐methoxysecoisolariciresinols (**12a/b**), and (±)‐5,5′‐dimethoxysecoisolariciresinols (**13a/b**) were prepared as described (Hwang et al., 2020).

### 4.2 PinZ expression and purification

Construction of an expression vector for hexa‐histidine‐tagged NrPinZ has been described previously (Allemann et al., 2025). The full‐length *pinZ* coding sequences from *N. aromaticivorans* F199 and *S. lignivorans* SYK‐6 were synthesized and cloned (GenScript) into the pET‐28a(+) backbone with an in‐frame N‐terminal hexa‐histidine tag (Novagen), with the resulting plasmids transformed into One Shot® BL21 Star™ (DE3) competent *E. coli* as described in Allemann et al. (2025). The heterologously expressed hexa‐histidine‐tagged NrPinZ, NaPinZ, and SlPinZ were purified on a POROS™ 20 MC metal chelate affinity column (Allemann et al., 2025).

### 4.3 PinZ enzyme assays

All PinZ assays were performed with racemic ligballinols (**1a/b**), pinoresinols (**2a/b**), medioresinols (**3a/b**), and syringaresinols (**4a/b**) as substrates. Assays consisted of buffer (20 mM Tris–HCl, pH 7.0, 185 μL), substrates (5 mM, 20 μL), NADPH (10 mM, 40 μL), and PinZ (5 μL, 18 ng) in a total volume of 250 μL. Reactions were incubated at 30°C for 10 min, stopped by addition of glacial acetic acid (10 μL), extracted with ethyl acetate (2 × 0.5 mL), and dried *in vacuo*. For reversed‐phase chromatography, dried assay extracts were resuspended in 50 μL methanol. A 10 μL aliquot of each reaction was injected. For corresponding chiral separations, dried extracts were resuspended in 100 μL ethanol, and 80 μL aliquots were injected. Assays were also carried out in the presence of NADH (10 mM, 40 μL) as described above for NADPH; however, no enzymatic activity was observed.

For steady‐state kinetic analyses with racemic pinoresinols (**2a/b**), medioresinols (**3a/b**), and syringaresinols (**4a/b**), assays were performed using 10 different substrate concentrations (0.5 to 400 *μ*M) and 6–10 ng of NrPinZ, NaPinZ, or SlPinZ. Reactions were carried out in triplicate and analyzed by reversed‐phase HPLC as described below. The Hyperbola function in Origin was used to determine the Michaelis–Menten kinetics parameters (*K*_m_ and *V*_max_) (Allemann et al., 2025).

### 4.4 High performance liquid chromatographic separations

HPLC separations were performed on an Alliance 2690 HPLC system (Waters, Milford, MA) equipped with UV–Vis diode‐array detector (Model 2990, Waters), with detection at 280 nm. Reversed‐phase separations used a Symmetry Shield RP_18_ column (150 × 3.5 mm, Waters) eluted with A (3% acetic acid in H_2_O) and B (acetonitrile) at a flow rate of 1 mL/min as follows: 95:0 from 0 to 2 min followed by linear gradients from 95:0 to 85:15 in 3 min, 85:15 to 60:40 in 25 min, 60:40 to 5:95 in 5 min after which the column was re‐equilibrated to the starting conditions.

Chiral‐phase chromatographic separations of (±)‐medioresinols (**3a/b**), (±)‐syringaresinols (**4a/b**), (±)‐5‐methoxylariciresinols (**7a/b**), (±)‐5′‐methoxylariciresinols (**8a/b**), and (±)‐5,5′‐dimethoxylariciresinols (**9a/b**) were carried out on a Chiralcel OD column (250 × 4.6 mm, Chiral Technologies, West Chester, PA) eluted with absolute EtOH at a flow rate of 0.5 mL/min. Chiral separations of (+)‐ and (−)‐enantiomers of pinoresinols (**2a** and **2b**), lariciresinols (**6a** and **6b**), and secoisolariciresinols (**11a** and **11b**) used a Chiralcel OC column (250 × 4.6 mm) eluted with EtOH‐hexanes (8:2, v/v) at a flow rate of 0.2 mL/min (Allemann et al., 2025).

### 4.5 Molecular modeling of NrPinZ and plant PLRs with racemic and enantiomeric substrates

Predicted models of NrPinZ and PLR_Tp2 were generated from their respective sequences with the diffusion‐based deep learning algorithm Boltz‐2 (Passaro et al., 2025). Since Boltz‐2 allows for the co‐folding of proteins with substrates and cofactors, both enzymes were folded in the presence of NADPH. Twenty independent predicted models for each enzyme were generated and ranked by their confidence scores [a weighted ranking derived from both pLDDT (predicted local distance difference test) and ipTM (interface predicted template modeling) scores]. Although Boltz‐2 is capable of co‐folding with two or more cofactors/substrates, attempts to co‐fold NrPinZ and PLR‐Tp2 with both NADPH and either (+)‐ or (−)‐pinoresinol gave mixed isomers of the substrates such that obtaining reasonable ternary complexes was neither reproducible nor consistent. Molecular docking receptor models of NrPinZ‐NADPH and PLR_Tp2‐NADPH were therefore generated with ICM‐Pro v3.6‐3a (Abagyan et al., 1994; Abagyan & Totrov, 1994) from the top‐ranked Boltz‐2 models by first converting the PDB files into ICM objects with optimization of hydrogen atom placement. Atom types and charges are assigned based on residue templates, and the ligands are assigned a protonation state using an internal machine learning pKa model, with partial charges based upon that state. An initial position for the substrate binding site was determined using the ICM pocketfinder routine (An et al., 2005). The models were then subjected to multiple docking runs with (+)‐ and (−)‐pinoresinols (**2a** and **2b**), medioresinols (**3a** and **3b**), and syringaresinols (**4a** and **4b**). Poses were extracted as PDB files for further analysis. All structural Figures were generated with Pymol v2.6.0 (Schrodinger).

MD simulations were performed in triplicate for 500 ns on the top‐ranked NrPinZ‐NADPH and PLR_Tp2‐NADPH binary complexes generated by Boltz‐2, and the NrPinZ‐NADPH‐pinoresinol and PLR_Tp2‐NADPH‐pinoresinol ternary complexes from docking. The models were prepared with Maestro (Schrodinger) using the OPLS3e force field (Roos et al., 2019), and the pre‐defined TIP3P water model (Jorgensen et al., 1983) was used to build the system. The overall charges of the models were neutralized with Na^+^ and Cl^−^ ions, and 0.15 M NaCl was added prior to building the system. The systems were minimized prior to the final 100 ns production step run at 300 K and 1 Atm pressure, using the Nosé–Hoover chain coupling scheme for temperature control and the Martyna–Tuckerman–Klein chain coupling scheme with a coupling constant of 2.0 ps for pressure control (Martyna et al., 1992). Non‐bonded forces were calculated using an r‐RESPA integrator. The MD simulations were performed using Desmond (Bowers et al., 2006) in the Schrodinger 2019‐2 release, and the trajectories were saved at 50 ps intervals for analysis. Analyses were carried out with Maestro using the Simulation Interactions Diagram and Simulation Event Analysis widgets. Maestro and Desmond were run on the SHERLOCK 3.0 HPC cluster at Stanford University.

## AUTHOR CONTRIBUTIONS

**Clyde A. Smith:** Conceptualization; methodology; software; data curation; supervision; resources; formal analysis; visualization; writing – review and editing; writing – original draft; investigation; funding acquisition. **Diana L. Bedgar:** Methodology; resources. **Michael A. Costa:** Methodology; data curation; resources. **Syed G. A. Moinuddin:** Methodology; validation; data curation. **Marco N. Allemann:** Methodology; data curation; resources. **Joshua K. Michener:** Conceptualization; investigation; partial funding acquisition; resources; formal analysis; project administration; supervision; data curation. **Laurence B. Davin:** Conceptualization; investigation; partial funding acquisition; writing – original draft; writing – review and editing; visualization; validation; methodology; formal analysis; project administration; supervision; data curation; resources. **Norman G. Lewis:** Conceptualization; investigation; partial funding acquisition; writing – original draft; methodology; validation; visualization; writing – review and editing; formal analysis; project administration; data curation; supervision; resources.

## Supporting information


**DATA S1.** Sequences used for docking.
**DATA S2.** NrPinz and PLRTp2 Boltz models docking poses.

## Data Availability

The data that support the findings of this study are available from the corresponding author upon reasonable request.
