# Supplementary material for: Molecular basis and biological relevance of bacterial and plant pinoresinol/lariciresinol reductase specificities
Source: Protein Sci. 2026 Jan 20;35(2):e70436. doi: 10.1002/pro.70436 (PMC12817470; doi:10.1002/pro.70436)
Supplement: Supplementary file 1 — DATA S1. Sequences used for docking. DATA S2. NrPinz and PLRTp2 Boltz models docking poses. [file PRO-35-e70436-s001.zip › Supplementary File 1.docx]

Sequences used:

>IiPLR1

MRENNSGEKTRVLVVGGTGTMGRRIVRACLAEGHETYVLQQPETRVDIEKVQLLYSYKRLGARLIEASFSDHQSLVSAVKQVDIVVAAMSGVHFRSHSILVQLKLVEAIKEAGNIKRFLPSEFGMDPSRMGHAMPPGRETFDQKLEVRNAIEAAGIPHTYVVGACFAAYFAGNLSQMGTLIPPKKKVNIYGDGNVKVVYVDEDDIAEYTAKTLDDPRTINKTVYVRPTENVLTQMELVQIWEKLTGKELEKTNISANDFLADIEDKEIPHQAGLGHFYHIFYEGCLTDHEVGDDEEASKLYPDVKYTRMDEYLKIFL

>PLR_Tp1

MDKKSRVLIVGGTGYIGKRIVNASISLGHPTYVLFRPEVVSNIDKVQMLLYFKQLGAKLIEASLDDHQRLVDALKQVDVVISALAGGVLSHHILEQLKLVEAIKEAGNIKRFLPSEFGMDPDIMEHALQPGSITFIDKRKVRRAIEAASIPYTYVSSNMFAGYFAGSLAQLDGHMMPPRDKVLIYGDGNVKGIWVDEDDVGTYTIKSIDDPQTLNKTMYIRPPMNILSQKEVIQIWERLSEQNLDKIYISSQDFLADMKDKSYEEKIVRCHLYQIFFRGDLYNFEIGPNAIEATKLYPEVKYVTMDSYLERYV

>PLR_Tp2

MEESSRVLIVGGTGYIGRRIVKASIALGHPTFILFRKEVVSDVEKVEMLLSFKKNGAKLLEASFDDHESLVDAVKQVDVVISAVAGNHMRHHILQQLKLVEAIKEAGNIKRFVPSEFGMDPGLMEHAMAPGNIVFIDKIKVREAIEAASIPHTYISANIFAGYLVGGLAQLGRVMPPSEKVILYGDGNVKAVWVDEDDVGIYTIKAIDDPHTLNKTMYIRPPLNILSQKEVVEKWEKLSGKSLNKINISVEDFLAGMEGQSYGEQIGISHFYQMFYRGDLYNFEIGPNGVEASQLYPEVKYTTVDSYMERYL

>PLR_Fi1

MGKSKVLIIGGTGYLGRRLVKASLAQGHETYILHRPEIGVDIDKVEMLISFKMQGAHLVSGSFKDFNSLVEAVKLVDVVISAISGVHIRSHQILLQLKLVEAIKEAGNVKRFLPSEFGMDPAKFMDTAMEPGKVTLDEKMVVRKAIEKAGIPFTYVSANCFAGYFLGGLCQFGKILPSRDFVIIHGDGNKKAIYNNEDDIATYAIKTINDPRTLNKTIYISPPKNILSQREVVQTWEKLIGKELQKITLSKEDFLASVKELEYAQQVGLSHYHDVNYQGCLTSFEIGDEEEASKLYPEVKYTSVEEYL

KRYV

>LuPLR1

MGRCRVLVVGGTGYIGKRIVKASIEHGHDTYVLKRPETGLDIEKFQLLLSFKKQGAHLVEASFSDHESLVRAVKLVDVVICTVSGAHSRSLLLQLKLVEAIKEAGNVKRFIPSEFGMDPARMGDALEPGRETFDLKMVVRKAIEDANIPHTYISANCFGGYFVGNLSQLGPLTPPSDKVTIYGDGNVKVVYMDEDDVATYTIMTIEDDRTLNKTMYFRPPENVITHRQLVETWEKLSGNQLQKTELSSQDFLALMEGKDVAEQIVIGHLYHIYYEGCLTNFDIDADQDQVEASSLYPEVEYTRMKDYLMIYL

>NrPinZ

MTRIVITGASGNYGRGVADALVAMGRAADLILITRKPEKLAERADQGCTVRQGDFDHPATLPQAMAGGDVLLLISGTRVGARVVQHKAAIDAAVAAGLRHIVYTSFIGIDDPANPAEVRHDHIETERLIRASGLAFTMLRDAHYADAMLLMAGPQVMQSGKWFANAGQGREAMVWRDDCIASAVAVLTTPGHENRIYNITGPELQTFAEVAAIMAEVTGCPVDYVDLDDDAQYALFDGLGIPRRPVDDQTVAGVPWNSDDMVTFGRAIREGFLEICTDDVERLTGRPARSTRAMVEANVAMLRAAAGR
